# Deletion C-terminal thioesterase abolishes melanin biosynthesis, affects metabolism and reduces the pathogenesis of *Fonsecaea monophora*

**DOI:** 10.1371/journal.pntd.0010485

**Published:** 2022-06-13

**Authors:** Minying Li, Huan Huang, Jun Liu, Xiaohui Zhang, Qian Li, Dongmei Li, Mingfen Luo, Xiaoyue Wang, Weiying Zeng, Jiufeng Sun, Hongfang Liu, Liyan Xi

**Affiliations:** 1 Dermatology Hospital, Southern Medical University, Guangzhou, China; 2 Guangdong Clinical College of Dermatology, Anhui Medical University, Guangzhou, China; 3 Department of Microbiology-Immunology, Georgetown University Medical Center, Washington, District of Columbia, United States of America; 4 Guangdong Provincial Institute of Public Health, Guangdong Provincial Center for Disease Control and Prevention, Guangdong, Guangzhou, China; 5 Department of Dermatology, Sun Yat-sen Memorial Hospital, Sun Yat-Sen University, Guangzhou, China; Universidad de Antioquia, COLOMBIA

## Abstract

Dematiaceous *Fonsecaea monophora* is one of the major pathogens of chromoblastomycosis. It has been well established that melanization is catalyzed by the type I polyketide synthase (PKS) in *F*. *monophora*. Multidomain protein Type I PKS is encoded by six genes, in which the last enzyme thioesterase (TE) catalyzes the cyclization and releases polyketide. Two PKS genes AYO21_03016 (*pks1*) and AYO21_10638 have been found in *F*. *monophora* and both PKS loci have the same gene arrangement but the TE domain in AYO21_10638 is truncated at 3’- end. TE may be the key enzyme to maintain the function of *pks1*. To test this hypothesis, we constructed a 3’-end 500 bp deletion mutant of AYO21_03016 (*Δpks1-TE-C500*) and its complemented strain. We profiled metabolome of this mutant and analyzed the consequences of impaired metabolism in this mutant by fungal growth *in vitro* and by pathogenesis *in vivo*. Compared with wild-type strain, we found that the mutant repressed *pks1* expression and other 5 genes expression levels were reduced by more than 50%, perhaps leading to a corresponding melanin loss. The mutant also reduced sporulation and delayed germination, became vulnerable to various environmental stresses and was less resistance to macrophage or neutrophil killings *in vitro*, and less virulence in mice footpad model. Metabolomic analysis indicated that many metabolites were remarkably affected in *Δpks1-TE-C500*, in particular, an increased nicotinamide and antioxidant glutathione. In conclusion, we confirmed the crucial role of C-terminal TE in maintaining fully function of *pks1* in *F*. *monophora*. Deletion of TE negatively impacts on the synthesis of melanin and metabolites that eventually affect growth and virulence of *F*. *monophora*. Any potential inhibitor of TE then could be a novel antifungal target for drug development.

## Author summary

*F*. *monophora* is a fungal pathogen that causes chromoblastomycosis. Melanin of *F*. *monophora* was synthesized through PKS in which TE is the last enzyme to catalyze the cyclization and release polyketide. Few studies have investigated the effect of TE on the metabolism and pathogenesis of *F*. *monophora*. In this study, TE deletion leads to albino phenotype, decreases the expression of other domains of the *pks1*, and reduces biosynthesis of metabolites. The *Δpks1-TE-C500* strain exhibits a changed morphogenesis and becomes less resistant to various environmental stresses. *In vitro* study, the *Δpks1-TE-C500* strain is avirulent and less resistant to macrophages and neutrophils. In conclusion, we demonstrate that the 500 bp C-terminal of TE is essential for the function of *pks1*, perhaps through its effects on melanin and metabolites to regulate the growth and virulence of *F*. *monophora*. Data from this study could inspire an exploration in development of clinical therapy for CBM.

## 1 Introduction

Chromoblastomycosis (CBM) is a chronic granulomatous infection caused by dematiaceous fungi invading the skin and subcutaneous tissue, which can infect healthy people in tropical and temperate zones. The disease is endemic worldwide and is considered as a neglected tropical disease (NTD) in 2017 [1]. *F*. *monophora* is one of the most common pathogens for chromoblastomycosis. Recently, we have reanalyzed the ITS rRNA sequences of causative strains previously collected in southern China and found that more than 80% of morphologically identified *Fonsecaea pedrosoi* in the past are actually *F*. *monophora* [2]. A number of studies have demonstrated that *F*. *monophora* exhibits neurotropism or causes primary brain infection without skin damage [3,4].

Little is known about the pathogenic traits of *F*. *monophora* and their impact in host immune defense response. Studies have shown that melanin on the fungal cell wall is a vital virulence factor in dematiaceous fungi because of its distinct chemical and biological attributes such as negative charge, hydrophobicity, acid resistance, and heat resistance [5]. The ability of resistance to external physical and chemical stresses confers melanin a protection from the host defense response, which has been confirmed with melanin-deficient strains of *Cryptococcus neoformans* [6], *F*. *pedrosoi* [7] and *Exophiala dermatitidis* [8]. In our previous study, we also found that albino *F*. *monophora* CBS125149 was more sensitive to environmental stresses and less virulent in mice model, compared with pigmented *F*. *monophora* CBS122845 [9,10].

Melanin synthesis in *F*. *monophora* is mainly accomplished through the 1,8-dihydroxynaphthalene (DHN) pathway [11]. Type I Polyketide synthase (PKS) is responsible for the first step of the biosynthetic DHN pathway. The multi-domain PKS is an enzyme complex [12], consisting of starter unit-ACP transacylase (SAT), β-ketoacyl synthase (KS), acetyl transferase (AT), dehydratase (DH), acyl carrier proteins (ACP) and thioesterase (TE). In our previous study, two homologous polyketide synthase genes, AYO21_03016 and AYO21_10638 were found in *F*. *monophora*. Both genes have conserved functional domains through InterProScan 4.8 (http://www.ebi.ac.uk/interpro/search/seque-nce/) analysis with default settings (**S1 Fig**). Since the albino strain can only be obtained by fully deletion of entire AYO21_03016 gene, but not AYO21_10638 [13], we thus speculated that AYO21_03016 (*pks1*) is the dominate gene for melanin synthesis in *F*. *monophora*. Subsequently, we compared the gene complex structures between AYO21_03016 and AYO21_10638, and found that the size of the TE gene was different between two gene complexes. As shown in **S1 Fig**, TE gene of AYO21_03016 is longer at its 3’- end than AYO21_10638, which leaves a protein with 132 amino acids truncation at C-terminus of TE in AYO21_03106. TE domain catalyzes the last step of polyketides synthesis for polyketide cyclization and release through a canonical catalytic triad consisting of Ser-His-Asp residues [14]. Sequence analysis revealed that the TE domain in AYO21_03016, likes many other PKS, contains these three residues at S72, D106 and H231 (**S2 Fig**), but the truncated TE in AYO21_10638 has no D106 and H231 residues, which can make TE loss of function. Therefore, TE domain may be necessary to maintain the function of PKS.

Polyketide products possess diverse architectures and biological functions. PKS biosynthesis even shares several steps with fatty acid synthesis, in which TE is an important link between PKSs and fatty acid synthases [15]. As PKS is also involved in the melanin and metabolite synthesis pathway, it is speculated that TE on *pks1* may also affect the resistance of *F*. *monophora* to external stress as well as virulence, and could be a potential drug target for *F*. *monophora* infection. In this study, we aimed to investigate the function of TE by characterizing the TE gene on *pks1* and exploring the mechanism of the host’s immune response to *F*. *monophora*.

## 2 Materials and methods

### Ethics statement

This study protocol was approved by the Ethics committee of the Dermatology Hospital, Southern Medical University (ethics amendment dated 7 March 2018, approval number 2018002. All experiments in this study were conducted according to internationally accepted standards and regulations on the administration of experimental animals in China (8/1/2011 C-WISC).

### 2.1 Strains, growth conditions, targeted gene deletion, and complementation analysis

**Fig 1:**
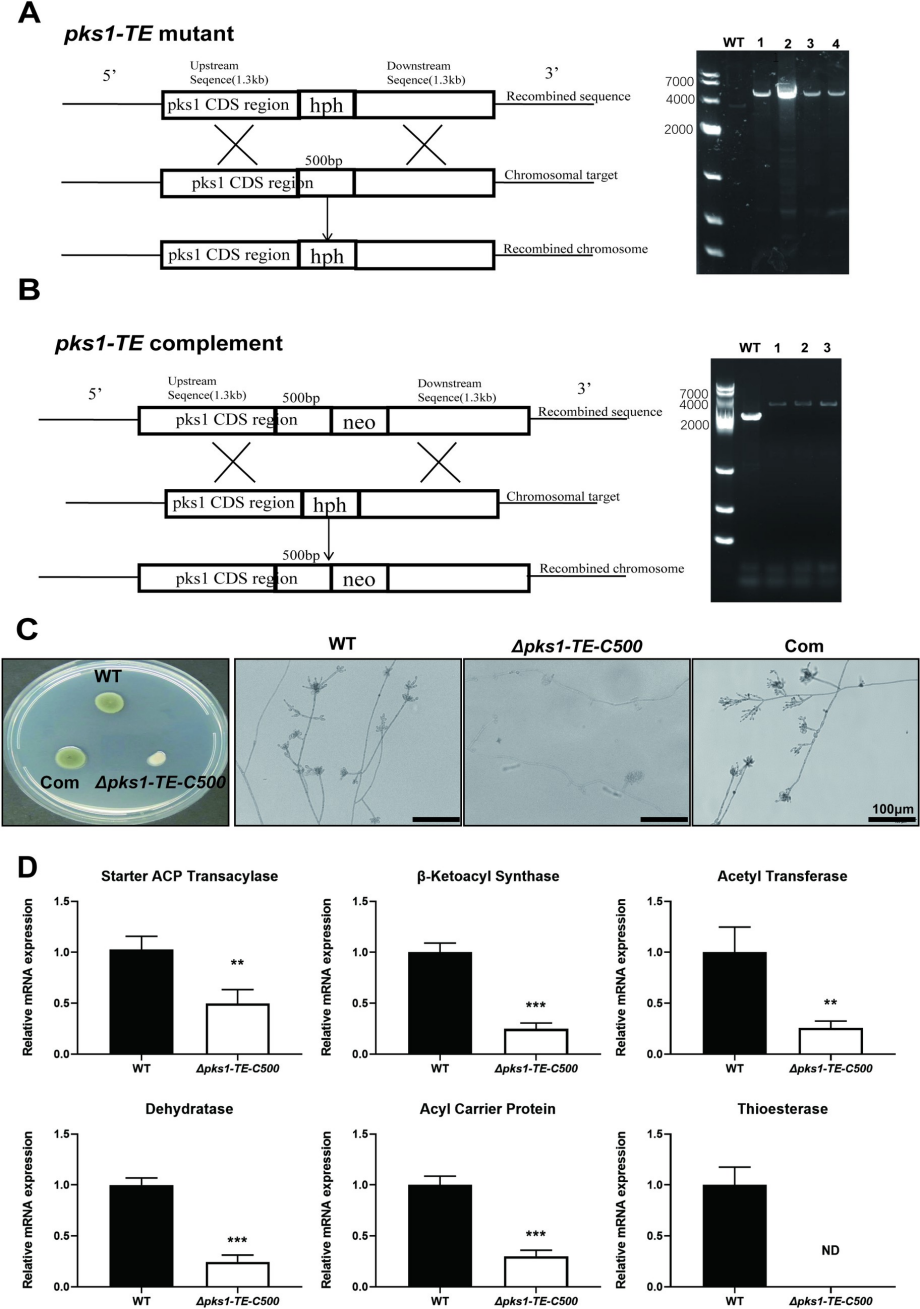
Construction and verification of the 500 bp C-terminal of TE deletion and complemented strain of *F*. *monophora*. (A) Schematic diagram of gene deletion. The resistance gene (hph) replacement strategy was used to destruct the 500 bp C-terminal of TE in *F*. *monophora*. Through homologous recombination, overlapping DNA fragments of the hygromycin resistance gene cassette (hph) were used for gene replacement. PCR identification analysis of the wild-type and deletion mutant strains. (B) Schematic diagram of gene complementation. Another resistance gene (neo) replacement strategy was used to complement the 500 bp C-terminal of TE in *F*. *monophora*. Through homologous integration, overlapping DNA fragments of the neomycin resistance gene cassette (neo) were used for gene replacement. PCR identification analysis of the wild-type and complementation strain. (C) Colony morphology and microscopic morphology examination of the wild-type and mutant strain. (D) The mRNA expression of conserved functional domains of AYO21_03016 protein in *F*. *monophora* were detected, including starter ACP transacylase (SAT), β-ketoacyl synthase (KS), acetyl transferase (AT), dehydratase (DH), acyl carrier protein (ACP) and thioesterase (TE). TE gene expression was negative in *Δpks1-TE-C500* and other 5 genes (SAT, KS, AT, DH, ACP) were significantly less expressed than WT. All statistical analysis were performed using two-tailed t-test (**, P<0.01; ***, P<0.001; ND means not detected, relative mRNA expression = 0). All the assays were performed in triplicate.

Strains and plasmids used in this study are listed in **Table 1**. *F*. *monophora* SUMS0310 (CBS269.37) was used as the wild-type (WT) strain in all experiments. Transformation was performed using the *Agrobacterium* tumefaciens-mediated transformation (ATMT) method [16]. The *Δpks1-TE-C500* was generated by transforming *F*. *monophora* with plasmids to delete the terminal 500 bp region of the *pks1*, followed by selection with hygromycin (**Fig 1A**). The complemented *Δpks1-*Com strain was generated by transforming the *Δpks1-TE-C500* with the *pks1-*TE-C-neo-pBHt2 plasmid and selecting for geneticin-resistant transformants (**Fig 1B**). The plasmids used for *pks1* TE targeted deletion and complementation, *pks1* TE-hph-pBHt2 and *pks1-*TE-C-neo-pBHt2, were constructed by In-Fusion HD Cloning Kit (Takara, Japan) according to the manufacturer’s instructions. For *pks1*-TE-hph-pBHt2 vector, PCR was used to obtain the four fragments: the 326 bp fragment upstream (primers 1 and 2) (**Table 1**), the 940 bp fragment downstream (primers 1 and 2) containing XhoI cleavage site fragment amplified from pBHt2 plasmid (Addgene plasmid # 104175), a 1300 bp fragment (primers *pks1-*TE arm2. FOR and *pks1-*TE arm2. REV) coding sequence, and a 1300 bp fragment (primers *pks1-*TE arm1. FOR and *pks1-*TE arm1. REV) (**S1 Table**) downstream of the *F*. *monophora* AYO21_03016 gene. The fragment containing XhoI cleavage site was inserted into SacII and AsiSI-restricted pBHt2 plasmid using In-Fusion HD Cloning Kit to obtain pBHt2-XhoI. As for *pks1*-TE-hph-pBHt2 vector, two AYO21_03016 gene homologous flank ranges were cloned to pBHt2-XhoI by the similar strategy. To construct TE complementation vector (*pks1*-TE-C-neo-pBHt2), fragment containing geneticin resistance gene was synthesized and inserted into XhoI and AflII-restricted *pks1*-TE-hph-pBHt2 by in-fusion method to generate *pks1-*TE-neo-pBHt2. PCR was used to obtain the 1300 bp fragment containing the 500 bp deletion coding sequence (primers *pks1*-Com arm3. FOR and *pks1*-Com arm3. REV), and inserted into SacI and BamHI-restricted *pks1-*TE-neo-pBHt2 by in-fusion method to obtain *pks-*TE-C-neo-pBHt2. PCR method was used to identify knockout or complemented *pks1* TE transformants with the primers (*pks1-*TE. FOR and *pks1-*TE. REV) (**S1 Table**). Primers *pks1-*TE. FOR and *pks1-*TE. REV are located at the left and right flank regions adjacent to the *pks1* TE homology respectively. The negative transformant was about 3.1 kb, the positive transformants were confirmed by 4.5 kb amplified fragment for *Δpks1-TE-C500* (**Fig 1A**), and 4.3 kb PCR fragment for complemented transformants (**Fig 1B**). Sanger sequencing also confirmed the transformants. The whole gene deletion strain *Δpks1* was also performed with the same strategy described above to compare the efficacy of *Δpks1-TE-C500* transformation. The overall transformation efficiency of *Δpks1-TE-C500* was higher than *Δpks1* (**S3 Fig**).

**Table 1:** Strains and plasmids used in this study.

Strain or plasmid	Genotype or characteristic
SUMS0310 (CBS269.37)	Wild-type of *F*. *monophora*
*Δpks1-TE-C500*	Knock out 500 bp C-terminal of TE domain of *F*. *monophora*
Com	Complemented strain of*Δpks1-TE-C500*
pBHt2	Hygromycin-resistant plasmid
pBHt2-XhoI	pBHt2 containing XhoI
*pks1-*TE-hph-pBHt2	Plasmid containing the TE gene and hygromycin resistance gene
*pks1-*TE-neo-pBHt2	Plasmid containing the TE gene and geneticin resistance gene
*pks1-*TE-C-neo-pBHt2	Plasmid containing the TE gene complemented fragment and geneticin resistance gene

### 2.2 RNA isolation and real time PCR

Total RNA of each sample was extracted using Trizell Reagent (Invitrogen, USA). Then, the RNA was reverse transcribed using RevertAid Master Mix (catalog no.M16325, Thermo, USA). Quantitative real-time reverse transcription-PCR (RT-qPCR) was carried out on the BioRad instrument (BioRadCFX96Touch, USA) by using PowerUp SYBR Green Master Mix (Thermo, USA), and the primers used are listed in **S1 Table**.

### 2.3 Metabolomics analysis

All strains were cultured on potato dextrose agar (PDA) at 25°C for 14 days. Subsequently, the strains were collected, and metabolites were extracted with 50% methanol buffer. In brief, 100 mg of sample was extracted with pre-cooled 50% methanol, homogenized for 1 min, and incubated at room temperature for 10 min; the extraction mixture was then stored overnight at -20°C. After centrifugation at 4,000 *g* for 20 min, the supernatants were transferred to new 96-well plates. The samples were stored at -80°C prior to Liquid chromatography-mass spectrometry (LC-MS) analysis. In addition, pooled Quality control (QC) samples were prepared by combining 10 μl of each extraction mixture. The identification and quantitative analysis of metabolites were carried out as described by Yukuo Li et al. [17].

### 2.4 Fungal growth, stress resistance

The growth rate and spore-producing ability of the mutants and WT were grown in PDA medium for 14 days at 25°C and 37°C, and radial growth was measured every 3 days over a period of 14 and 24 days. For each strain, 5 μl of conidial suspension in PBS containing 1×10^6^ conidia/mL was inoculated. Conidial number was counted as the number of spores per mm^2^after 14 days. Germination was examined with initial spores (1×10^6^) of each strain in 30 ml of Sabouraud Dextrose Broth (SDB) at 0, 12, 24 and 48 h after incubation at 25°C and 37°C. For the stress response of the strains, a 5 μl suspension containing 1×10^6^ conidia per ml was inoculated on PDA supplemented with KCl at 0.2, 0.4, and 0.6 M (salt stress), H_2_O_2_ at 0.5, 1, 2, and 2.5 mM, menadione at 15, 30, and 60 μM, SNAP (S-nitroso-N-acetylpenicillamine, a nitric oxide donor) at 0.225, 0.45 and 0.9 mM (oxidative stress) and sorbitol at 0.25, 0.5, and 1 M (osmotic stress). Exposures to 254 nm UV irradiation for 5, 10, and 15 min were also tested for UV resistance. All cultures were incubated for 14 days at 25°C.Based on references [10,18] and pre-experiments, the concentrations of KCl, H_2_O_2_, SNAP, menadione (VitK), and sorbitol were selected. The ability of melanin recovery in albino strains and pigmented strains were cultured on PDA with 50 mg/L tricyclazole at 25°C for 14 days. Tricyclazole is an inhibitor of pentaketide melanin biosynthesis (DHN pathway). Formation of melanin was tested by supplementation of PDA with 25 mg/l L-DOPA or 25 mg/l L-tyrosine in all strains. Concentrations of L-DOPA, L-tyrosine and tricyclazole were selected according to previous research [19]. The cultures were kept in the dark to withstand the autopolymerization of L-DOPA at 25°C for 14 days.

### 2.5 Evaluation of macrophage and neutrophil killing ability

RAW264.7 macrophages were infected with *F*. *monophora* conidia (multiplicity of infection (MOI) = 10:1, 10 conidia per cell) for 24 h at 37°C. The cells were incubated at 37°C with 5% CO_2_ for 24 h. Then, sterilized double-steamed water (1 mL) was used to release the spores phagocytosed by the cells. The diluted suspension was transferred to PDA plates and incubated at 25°C, and the number of colonies on each plate was counted after 7 days. For microscopy, infected macrophages were either fixed and prepared for Transmission electron microscopy (TEM) or stained with 5 mM Dil for 10 min, washed twice with PBS, fixed in 4% paraformaldehyde, and stained with calcofluor to examine morphogenesis. For TEM, cells were fixed with 2.5% glutaraldehyde for 2 h at 4°C. Then, they were washed in 0.1 mol/L PBS six times for 30 min each time, fixed with 1% osmic acid for 2 h, washed in 0.1 mol/L PBS three times for 10 min each time, and ethanol-dehydrated by sequential washing in 30%, 50%, 70%, 80%, 90%, 100% ethanol, 100% ethanol: 100% acetone (1:1), and 100% acetone for 10 min each time. The samples were embedded in white resin, and thin sections were examined with a JEM-1400 PLUS Transmission Electron Microscope (Japan Electron Optics Laboratory Co., Ltd., Japan).

Human neutrophils were isolated using PolymorphPrep density gradient media (1114683; PROGEN BioTEchnik GmbH, Heidelberg, Germany) according to the manufacturer’s protocol. Neutrophil fungicidal assay was performed with reference to a previous study [20]. In brief, purified neutrophils (1×10^6^) were infected with *F*. *monophora* conidia (MOI = 1:1) for 1 h or 2 h at 37°C under homogenization. Different MOIs were previously tested, which showed similar results; thus, a MOI of 1:1 was chosen. As a control, conidia were incubated under the same conditions without neutrophils. After incubation, an aliquot was taken and diluted in distilled water to induce neutrophil lysis without harming the fungi. The fungi were seeded on PDA and incubated for 7 days at 25°C for colony-forming unit (CFU) counting. The survival rate of the control group (fungi without neutrophils) was set as 100%.

Phagocytosis was determined by flow cytometry analysis according to Voyich et al. [21]. In brief, 2×10^5^ neutrophils were infected with the FITC-labeled conidia (2×10^6^) of different strains of *F*. *monophora* in RPMI 1640 media (10% FBS and 1% penicillin/streptomycin) for 1 h and 2 h at 37°C. Phagocytosis was stopped by the addition of ice-cold PBS. Samples were washed, taken up in cold PBS and 2% FBS, and analyzed with FACSAriaIII (Becton Dickinson). The mean fluorescence intensity per cell was calculated to estimate the amount of phagocytosed conidia.

### 2.6 Animal model

All animals were treated according to National Institutes of Health guidelines for the use of experimental animals. The study protocol was approved by the ethics committee of Dermatology Hospital, Southern Medical University, and conformed to the Guide for the Care and Use of Laboratory Animals. Female BALB/c mice (6–8 weeks old, 16 mice in each group). The mice were randomly divided into four groups: control group, wild-type strain, *pks1* TE mutant strain, and complemented strain group. The footpad was injected with 50 μl of the fungal solution containing 1×10^6^ fungal cells or PBS as the control in the plantar cushion. The thickness of the footpad was measured with the aid of a caliper. At 3, 5, 7, and 14 days after injection, the infected mice were sacrificed to obtain footpad samples for future analyses, including CFU counting, histopathological examination, RNA sequence analysis, and cytokine detection. For cytokine detection, Mouse Cytokine & Chemokine Panel 1 (eBioscience, San Diago, USA) was used according to manufacturer’s instructions. The footpad samples were put in the PBS with protease inhibitor, after homogenization, centrifuged (4°C, 16,000*g*, 10min) and took the supernatant for Th1 and Th17-related cytokines (IL-6, TNF-α, IL-1β, IL-17A, IL-22), and neutrophil-related chemokines (CCL2, CCL3, CXCL1, CXCL2) detection. The study was conducted according to the guidelines of the Declaration of Helsinki, and approved by the Institutional Review Board (or Ethics Committee) of Dermatology Hospital, Southern Medical University, Protocol 2018002 (7 March 2018).

### 2.7 Immunohistochemistry

To verify above suggestive neutrophil activation within WT-infected mice tissues, we then analyzed the inflammatory cells by immunohistochemistry using myeloperoxidase (MPO) antibody. Immunohistochemical staining was carried out as described previously [22]. Briefly, paraffin-embedded footpad tissues were sectioned at 3 μm. Tissue sections were deparaffinized and hydrated and incubated in 10% H_2_O_2_ for 10 minutes to block endogenous peroxidase. After sections were blocked with goat serum, the sections were incubated with MPO antibody (ab208670) overnight at 4°C. Subsequently, tissue sections were stained with horseradish peroxidase-coupled secondary antibody and detected by the Envision System. The assays were performed at least in triplicate.

#### 2.8.1 mRNA sequencing by Illumina HiSeq /Novaseq or MGI2000 and data analysis

Total RNA of each sample was extracted from infected footpads using Trizell Reagent. According to the previous method [23], total RNA of each sample was quantified and qualified by Agilent 2100/2200 Bioanalyzer (Agilent Technologies, Palo Alto, CA, USA), NanoDrop (Thermo Fisher Scientific Inc.). 1 μg total RNA was used for following library preparation. Next generation sequencing library preparations were constructed according to the manufacturer’s protocol. The poly(A) mRNA isolation was performed using Poly(A) mRNA Magnetic Isolation Module. The mRNA fragmentation and priming were performed using First Strand Synthesis Reaction Buffer and Random Primers. First strand cDNA was synthesized using ProtoScript II Reverse Transcriptase and the second-strand cDNA was synthesized using Second Strand Synthesis Enzyme Mix. The purified double-stranded cDNA by beads was then treated with End Prep Enzyme Mix to repair both ends and add a dA-tailing in one reaction, followed by a T-A ligation to add adaptors to both ends. Size selection of Adaptor-ligated DNA was then performed using beads, and fragments of ~ 400 bp (with the approximate insert size of 300 bp) were recovered. Each sample was then amplified by PCR using P5 and P7 primers, with both primers carrying sequences which can anneal with flow cell to perform bridge PCR and P5/ P7 primer carrying index allowing for multiplexing. The PCR products were cleaned up using beads, validated using a Qsep100 (Bioptic, Taiwan, China), and quantified by Qubit 3.0 Fluorometer (Invitrogen, Carlsbad, CA, USA). Then libraries with different indices were multiplexed and loaded on an Illumina HiSeq/Novaseq instrument according to manufacturer’s instructions (Illumina, San Diego, CA, USA) or a MGI2000 instrument according to manufacturer’s instructions (MGI, Shenzhen, China). Sequencing was carried out using a 2x150 paired-end (PE) configuration; image analysis and base calling were conducted by the HiSeq Control Software (HCS) + OLB + GAPipeline-1.6 (Illumina) on the HiSeq instrument, image analysis and base calling were conducted by the NovaSeq Control Software (NCS) + OLB + GAPipeline1.6 (Illumina) on the NovaSeq instrument, image analysis and base calling were conducted by the Zebeacall on the MGI2000 instrument. The sequences were processed and analyzed by GENEWIZ.

#### 2.8.2 Quality control & mapping

In order to remove technical sequences, including adapters, PCR primers, or fragments thereof, and quality of bases lower than 20, pass filter data of fastq format were processed by Cutadapt (V1.9.1) to be high quality clean data.

Reference genome sequences and gene model annotation files of relative species were downloaded from NCBI. Secondly, Hisat2 (v2.0.1) was used as index reference genome sequence. Finally, clean data were aligned to reference genome via software Hisat2 (v2.0.1).

#### 2.8.3 Differential gene expression and Gene Ontology (GO) and KEGG enrichment analysis

In the beginning transcripts in FASTA format are converted from known GFF annotation file and indexed properly. Then, with the file as a reference gene file, HTSeq (v0.6.1) estimated gene and isoform expression levels from the pair-end clean data.

Differential expression analysis used the DESeq2 Bioconductor package, a model based on the negative binomial distribution. he estimates of dispersion and logarithmic fold changes incorporate data-driven prior distributions, Padj of genes were settled. GOSeq (v1.34.1) was used identifying GO terms that annotate a list of enriched genes with a significant padj less than 0.05. And topGO was used to plot DAG. KEGG databases (Kyoto Encyclopedia of Genes and Genomes) is a collection of databases dealing with genomes, biological pathways, diseases, drugs, and chemical substances. We used scripts in house to enrich significant differential expression gene in KEGG pathways.

### 2.9 Statistical analysis

Data are presented as the mean ± SEM. Unpaired Student’s t-test with two-tailed P-values and ANOVA test was used for statistical analyses unless indicated otherwise (GraphPad Prism 5 software). In all tests, P-values less than 0.05 were considered statistically significant.

The numerical data used in all figures are included in **S1 Data**.

## 3. Results

### 3.1 C-terminal TE deletion down-regulates the *pks1*gene expression and causes albino phenotype in *F*. *monophora*

To determine the role of TE on *pks1*, particularly C-terminus of TE in metabolism and growth of *F*. *monophora*, C-terminal deletion strain *Δpks1-TE-C500* and complemented strain were successfully constructed (**Fig 1A and 1B**) and used to test loss-of-function phenotypes. After knocking out 500 bp C-terminal of TE domain, there are only 86 amino acids on the TE of AYO21_03016 (**S1 Fig**), without D106 and H231 residues, which can make TE loss of function. Melanin loss was clearly shown in *Δpks1-TE-C500* that exhibited small and white colony on PDA plate (**Fig 1C**). Under microscopy, the conidia and hyphae were also less pigmented in *Δpks1-TE-C500* strain (**Fig 1C**). Depigmentation in *Δpks1-TE-C500* was sustained despite of supplementation of melanin agonist L-DOPA or L-tyrosine (**S4 Fig**). Tricyclazole, a DHN melanin inhibitor, caused a de-coloration in WT but had no effect on *Δpks1-TE-C500*. At transcription level, TE gene expression was negative in *Δpks1-TE-C500* and the expression level of other 5 genes SAT, KS, AT, DH, ACP was reduced by more than 50%, compared with WT (**Fig 1D)**.

### 3.2 C-terminal TE deletion reduces *in vitro* growth and germination of *F*. *monophora*

**Fig 2:**
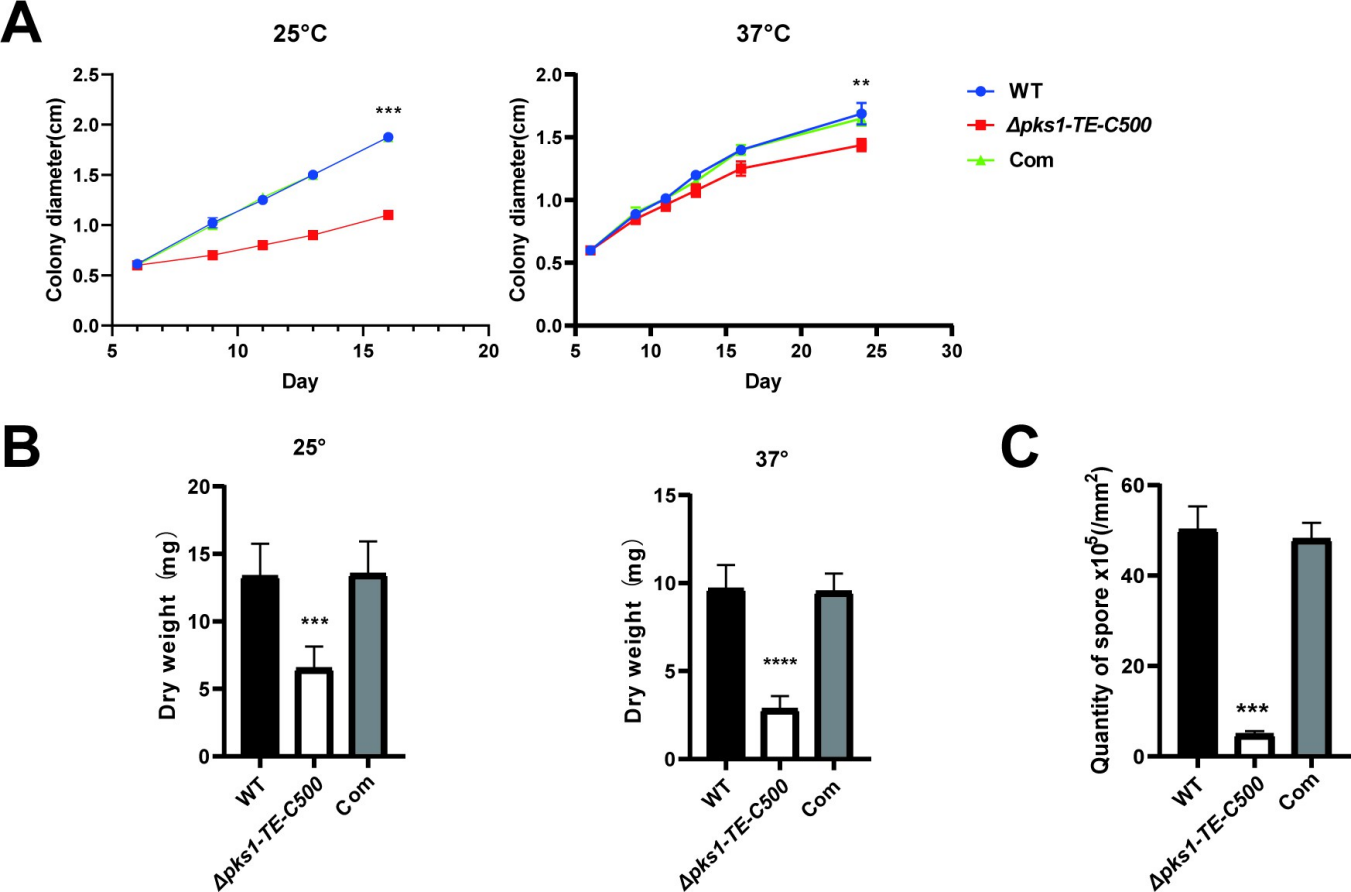
Effects of the 500 bp C-terminal of TE on the radial growth, biomass and conidiation of *F*. *monophora*. (A) The conidia of each strain were inoculated on PDA and incubated at 25°C and 37°C for 14 days. The radial growth rate of the *Δpks1-TE-C500* strain was significantly than that of the wild-type and Complemented strain. (B) After the growth of each strain on PDA for 14 days at 25°C and 37°C, the cells were collected, dried, and weighed. The *Δpks1-TE-C500* had a significantly lower biomass. (C) After the growth of each strain on PDA for 14 days, samples of fungi (1 cm^2^) were collected; the spores were separated, and the number of spores was counted. The *Δpks1-TE-C500* had fewer spore. All statistical analysis were performed using two-tailed t-test (**, P<0.01; ***, P<0.001; ****, P<0.0001).

To further understand the growth effects of a down-regulated *pks1*, growth rate, sporulation and germination of *F*. *monophora* were measured. We found that the colony diameter of *Δpks1-TE-C500* was significantly reduced, especially at 25°C when compared with the wild-type strain (**Fig 2A**). The biomass of 14 days growth of *Δpks1-TE-C500* was only half of wild-type and complemented strains at 25°C and 37°C (**Fig 2B**). In addition, the *Δpks1-TE-C500* strain exhibited an approximately 10-fold decrease in sporulation (**Fig 2C**) and showed a delayed germination. Compared with the germination course of wild-type and complemented strains in which germination began at 12 h and the long hyphae formed at 24 h, the mutant strain showed no germination at 12 h, started to germinate at 24 h and no hyphae were formed until 48 h (**S5 Fig**).

### 3.3 C-terminal TE deletion increases sensitivity of *F*. *monophora* to osmotic and oxidative stress

**Fig 3:**
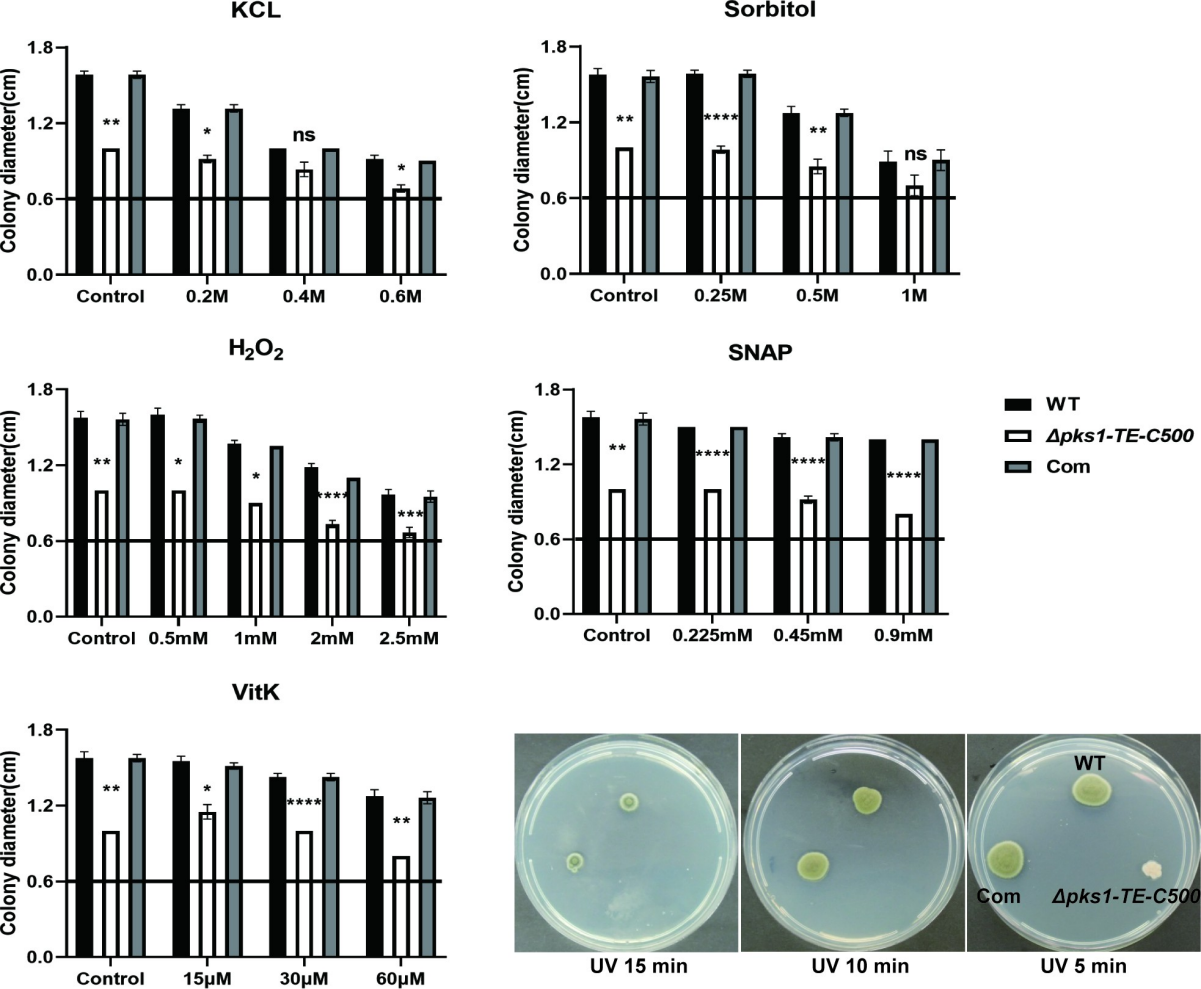
The 500 bp C-terminal of TE is involved in the stress response *in vitro*. In order to evaluate the resistance of *Δpks1-TE-C500*, all strains were inoculated on PDA with a 5 μl suspension containing 1×10^6^/ml conidia under specific conditions. For salt stress, 0.2, 0.4, and 0.6 M potassium chloride were added. For oxidative stress, 0.5, 1, 2, and 2.5 mM H_2_O_2_, 0.225, 0.45 and 0.9 mM SNAP, or 15, 30, and 60 μM vitamin K were added. For osmotic stress, 0.25, 0.5, and 1 M sorbitol were added. Then, the plates were incubated for 14 days at 25°C (The straight line represents the basic level on the fifth day (0.6cm)). For UV stress, the strains were irradiated with UV light for 5, 10, and 15 min. Compared to the wild-type, the growth of the *Δpks1-TE-C500* decreased significantly with the increase of stress factor concentration or UV irradiation time, especially oxidative stress and UV irradiation. Statistical significance was determined by ANOVA test (ns, not significant; *, P<0.05; **, P<0.01; ***, P<0.001; ****, P<0.0001).

Stress adaptation of TE mutant to salt stress and or oxidative stress were tested in the PDA plates containing different concentrations of KCl, H_2_O_2_, SNAP, menadione (VitK), and sorbitol. The data revealed that the mutant was highly sensitive to oxidative stress. The colony diameters of *Δpks1-TE-C500* are markedly reduced on PDA containing various concentrations of H_2_O_2_, SNAP, or VitK versus wild-type strain. The mutant strain also showed significant growth inhibition at various concentrations of KCl and sorbitol except 0.4 M KCl and 1 M sorbitol, and was easily killed by UV irradiation. When compared with mutant strain, the parental strain and complemented strain showed resistance to each of tested chemicals in a dose-dependent manner (**Fig 3**).

### 3.4 Deletion of C-terminal TE *F*. *monophora* alters secondary metabolites, lipid and polyketides metabolism

**Fig 4:**
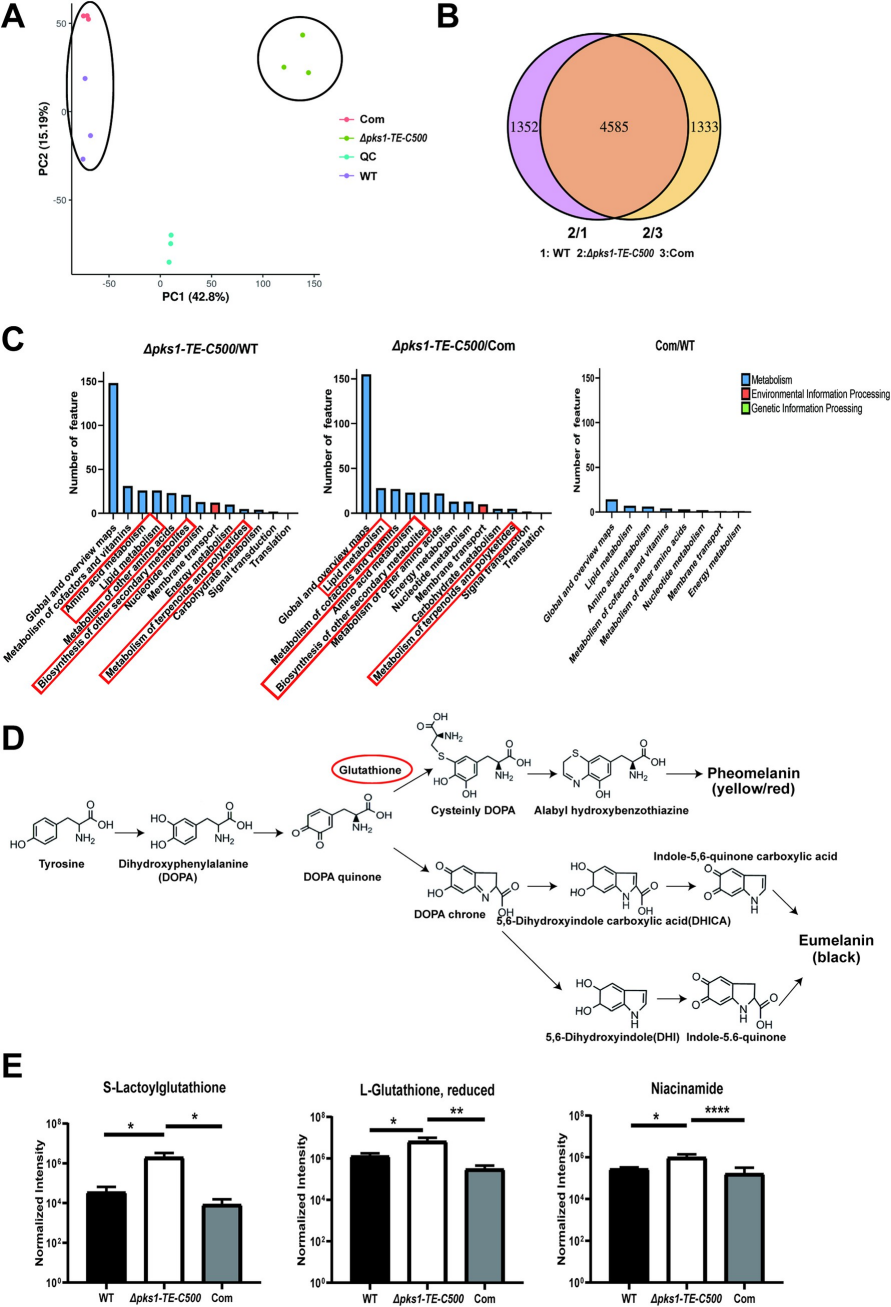
Effects of the 500 bp C-terminal of TE on metabolites. All strains were incubated at 25°C and collected after 14 days for the analysis of secondary metabolites. (A) PCA diagram of the identified metabolites of the WT and mutant strains. (B) Venn diagram of the WT and mutant strains. (C) KEGG pathway enrichment analysis of the WT and mutant strains. (D) The DOPA-melanin pathway. Glutathione (labeled by red circle) is implicated in the biogenesis of the pheomelanin. (E) The yields of L-glutathione (reduced), S-lactoylglutathione, and niacinamide were increased in the albino strain. All statistical analysis were performed using two-tailed t-test (*, P<0.05; **, P<0.01; ****, P<0.0001). There were three biological replicates in each experiment.

To understand the roles of *F*. *monophora* PKS TE domain on metabolism, metabolomic profiles of the C-terminal TE mutant *Δpks1-TE-C500* was compared with WT and complemented strain (**Fig 4**). In the principal component analysis (PCA) diagram, WT and the complemented strain were separated from the *Δpks1-TE-C500*, which implied that WT and the complemented strain were not similar to *Δpks1-TE-C500*. (**Fig 4A**). We found that deletion of C-terminal TE accounted for 23% differential metabolites between mutant and either WT or complemented strain, a total of 4585 metabolites (77%) were overlapped among three strains (**Fig 4B**). KEGG enrichment analysis showed that knockout *pks1* TE C-terminus specifically effected terpenoid and polyketide production, as evidenced by the 90% loss of gossypol and hydroxyspheroidenone and the 85% loss of okenone (**S2 Table and Fig 4C**). Lipid biosynthesis and amino acids metabolism were also influenced after knocking out TE **(Fig 4C)**. We also found that biosynthesis of other secondary metabolites such as cephalosporin_C and penitrem_D reduced by 90% as well in mutant strain (**S2 Table**). Accompanying with metabolites impairment as shown above, reduced form of L-glutathione, S-lactoylglutathione and niacinamide increased in the *Δpks1-TE-C500* versus those of the wild-type and complemented strain (**Fig 4E**), which could be a compensatory response to decreased polyketides. In DOPA-melanin pathway, glutathione is required for formation of pheomelanin (yellow or red), but not for eumelanin (black) (**Fig 4D**).

### 3.5 Deletion of C-terminal TE *F*. *monophora* increases phagocytotic killing

**Fig 5:**
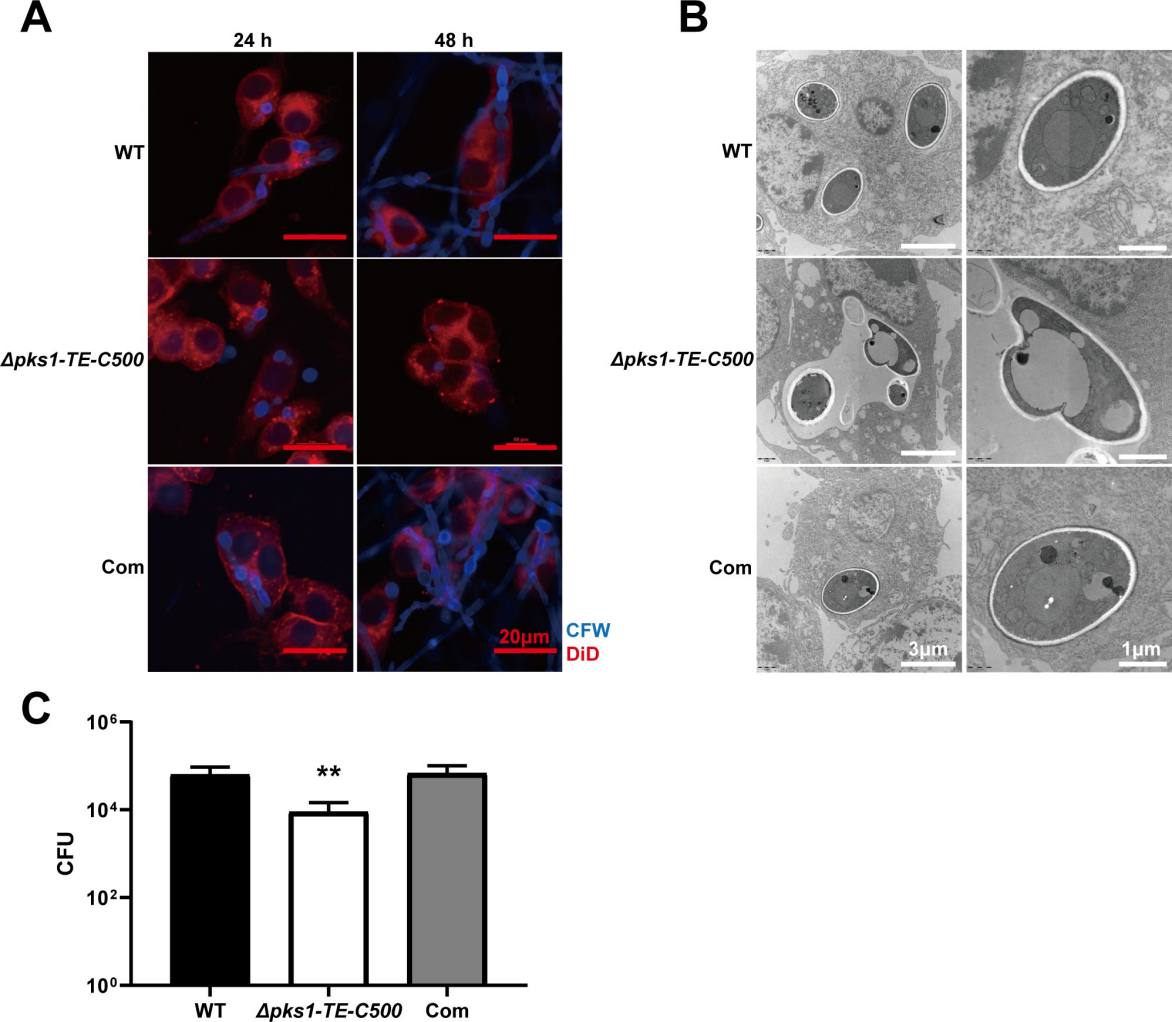
The 500 bp C-terminal of TE is essential for survival in macrophages. (A) The wild-type and *Δpks1-TE-C500* strain were co-cultured with RAW264.7 macrophages for 24 and 48 h. In comparison with the wild-type strain, the conidia of the *Δpks1-TE-C500* were more sensitive to cytotoxic activity and were more easily killed and cleared by macrophages. The wild-type hyphae penetrated the cells in 48 h. (B) TEM analysis after 24 h of co-culture. The wild-type and complemented strain maintained the integrity of the cell wall of the conidia after 24 h in cytoplasm, and macrophage mitochondria were swollen (black arrow). In contrast, the conidia of the *Δpks1-TE-C500* were deformed in phagosome, and the mitochondria were normal (white arrow). (C) Conidial survival was detected by counting CFU on PDA after the infected macrophages lyses. The number of surviving colonies of the *Δpks1-TE-C500* strain was much lower than that of the wild-type strain. The statistical analysis was performed using two-tailed t-test (**, P<0.01).

The consequence of melanin loss and decreased growth rate of the *Δpks1-TE-C500* in immune escape is then evaluated. All strains were co-cultured with macrophages (RAW264.7) to detect phagocytotic killing at 24 and 48 h after incubation. We found that WT conidia could be rapidly phagocytosed by the macrophages at 12 h and they started to germinate at 24 h and formed hyphae at 48 h within macrophages, causing immune escape (**Fig 5A**). The conidia of the mutant strain were also readily phagocytosed by macrophages at 24 h, however, the amount of conidia in the cells were significantly reduced by 48 h with no hyphae as most of the spores were killed and degraded. TEM indicated wild-type conidia maintained normal cell morphology inside the macrophages with the intact cell wall and organelles. The mutant conidia were in the phagosome at 24 h (**Fig 5B**). After 24 h of incubation, wild-type or mutant strain-infected macrophages were lysed for detecting fungal burden. Compared with the wild-type group, the mutant group exhibited sharply decreased CFU in the infected macrophages (**Fig 5C**). In addition, the mitochondria of wild-type and complemented strain-stimulated macrophages showed swelling and vacuolation (usually due to cell damage), which could not induce by the *Δpks1-TE-C500* strain.

### 3.6 Deletion of C-terminal TE *F*. *monophora* decreases virulence

**Fig 6:**
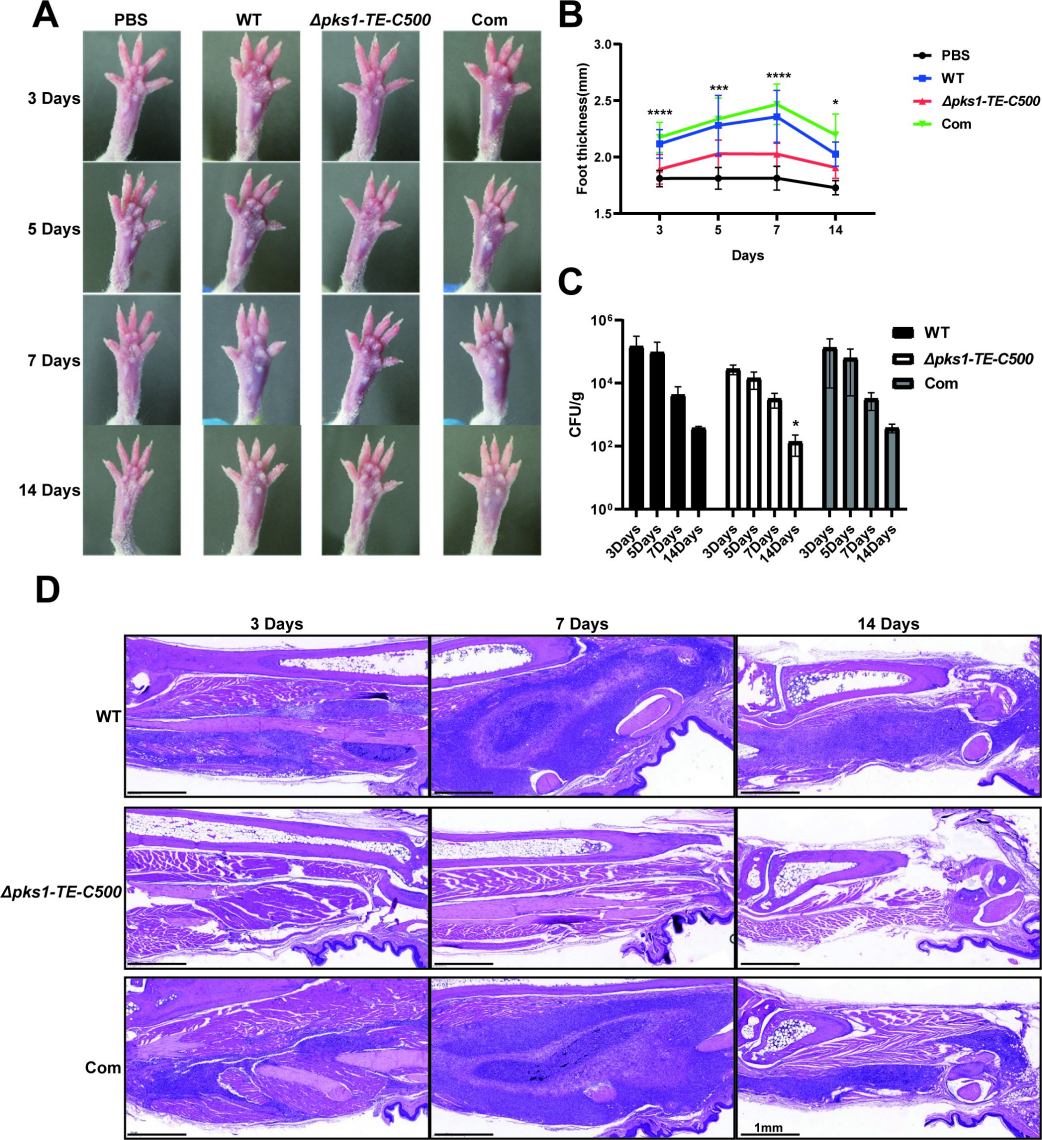
Effect of 500 bp C-terminal of TE deletion on virulence in the *F*. *monophora* infection mice model. The conidia (1×10^6^) of all strains injected into the footpads of mice to infect mice. (A and B) The footpad thickness of mice were photographed and measured 3, 5, 7 and 14 days after infection. The footpad thickness of mice in *Δpks1-TE-C500* group were significantly thinner than those of mice in the wild-type group and the complemented group in different stages. (C) The fungal burden of footpads was then detected. The number of CFU for the *Δpks1-TE-C500* was significantly reduced compared with that of the wild-type and complemented strain at 14 days. (D) Histopathology of footpads injected with all strains at different time points (day 3, 7, and 14). In comparison with footpads injected with the wild type and complemented strain, footpads injected with the *Δpks1-TE-C500* were less swollen, and the number of CFU in the tissues was significantly reduced. In addition, the number of inflammatory cells of *Δpks1-TE-C500* group in the pathological tissues was decreased. All statistical analysis were performed using two-tailed t-test, and the results were statistically significant (*, P<0.05; ***, P<0.001; ****, P<0.0001).

We next tested virulence of mutant on our previously-established murine paw infectious model. We found that the *Δpks1-TE-C500* strain is significantly less virulent than control strains (**Fig 6)**. The paw swelling and inflammation of mice infected with the *Δpks1-TE-C500* strain are significantly less severe than those of mice infected with the wild-type strain and complemented strain (**Fig 6A and 6B**), especially at 7 days. The fungal burden in the infected footpads of the *Δpks1-TE-C500* (136.5 CFU/g) is significantly less than those of the wild-type strain (374.7 CFU/g) and complemented strain (380 CFU/g) at 14 days (**Fig 6C**). The inflammatory response is overall weakly induced by *Δpks1-TE-C500*, when compared with abundant inflammatory foci over the injection site in the footpads of mice injected with the wild-type and complemented (**Fig 6D**), in which a large number of mixed inflammatory infiltrates and granulocytes are found.

### 3.7 The*Δpks1-TE-C500*-infected mice exhibited a weak pro-inflammatory cytokine response

**Fig 7:**
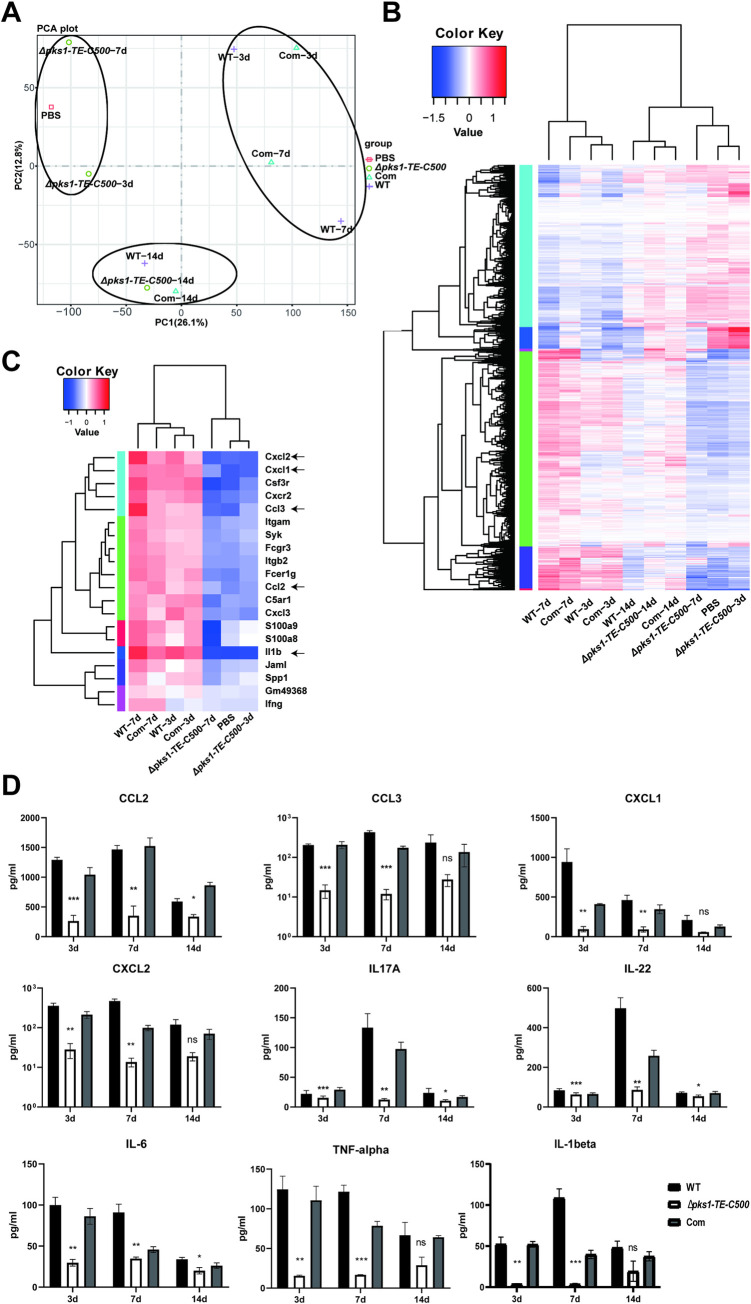
Association of 500 bp C-terminal of TE deletion with a lack of immune response. (A-C) RNA-seq analysis of the whole-genome transcript profiles of mice infected with all strains and PBS. (A) PCA showed the *Δpks1-TE-C500* group was relatively similar to the PBS group on the 3rd and 7th day. (B) Heatmap of the RNA-seq data for all groups. (C) Heatmap of gene expression in neutrophil chemotaxis in all groups. (D) Specific proinflammatory cytokines and neutrophil chemokines were significantly reduced in *Δpks1-TE-C500* group on the 3rd, 7th and 14th day after injection, compared with wild-type group. Statistical significance was determined by two-tailed t-test (**P<0.01; ***P<0.001; ****P<0.0001).

The cytokine profile revealed a strong neutrophil chemotaxis response in WT-infected footpads, which was consistent with granulocytes enhancement in histopathology. PCA analysis showed that the *Δpks1-TE-C500* group was relatively similar to the PBS group on the 3rd and 7th day, while WT group was alike to the complemented group (**Fig 7A**). The gene expression between the WT and the complemented strain at day 3 and day 7 were analogous in heatmap (**Fig 7B**) and volcano map (**S6 Fig**). GO enrichment analysis showed that the differentially expressed genes were significantly enriched in inflammatory response and neutrophil chemotaxis in WT versus *Δpks1-TE-C500*-infected footpads at day 3 and day 7 after infection (labeled with black arrow) (**S7A Fig**). KEGG enrichment demonstrated that the differential genes were enriched in chemokine signaling pathway and Th17 cell differentiation (labeled with black arrow) (**S7B Fig**). Heatmap of neutrophil chemotaxis indicated that gene expression in wild-type strain was higher than mutant strain (**Fig 7C**). Proinflammatory cytokines associated with Th17/Th1 response such as IL-6, TNF-α, and IL-1β, and neutrophil-recruiting chemokines including CCL2, CCL3, CXCL1, and CXCL2 were highly produced from day 3 to day 14 after WT infection. Avirulent *Δpks1-TE-C500* produced much low levels of the same panel of cytokines, especially at day 3 and day 7 after infection (**Fig 7D**). These results were consistence with RNA-seq data.

### 3.8 *Δpks1-TE-C500*-infected mice has less neutrophil recruitment in the footpads

**Fig 8:**
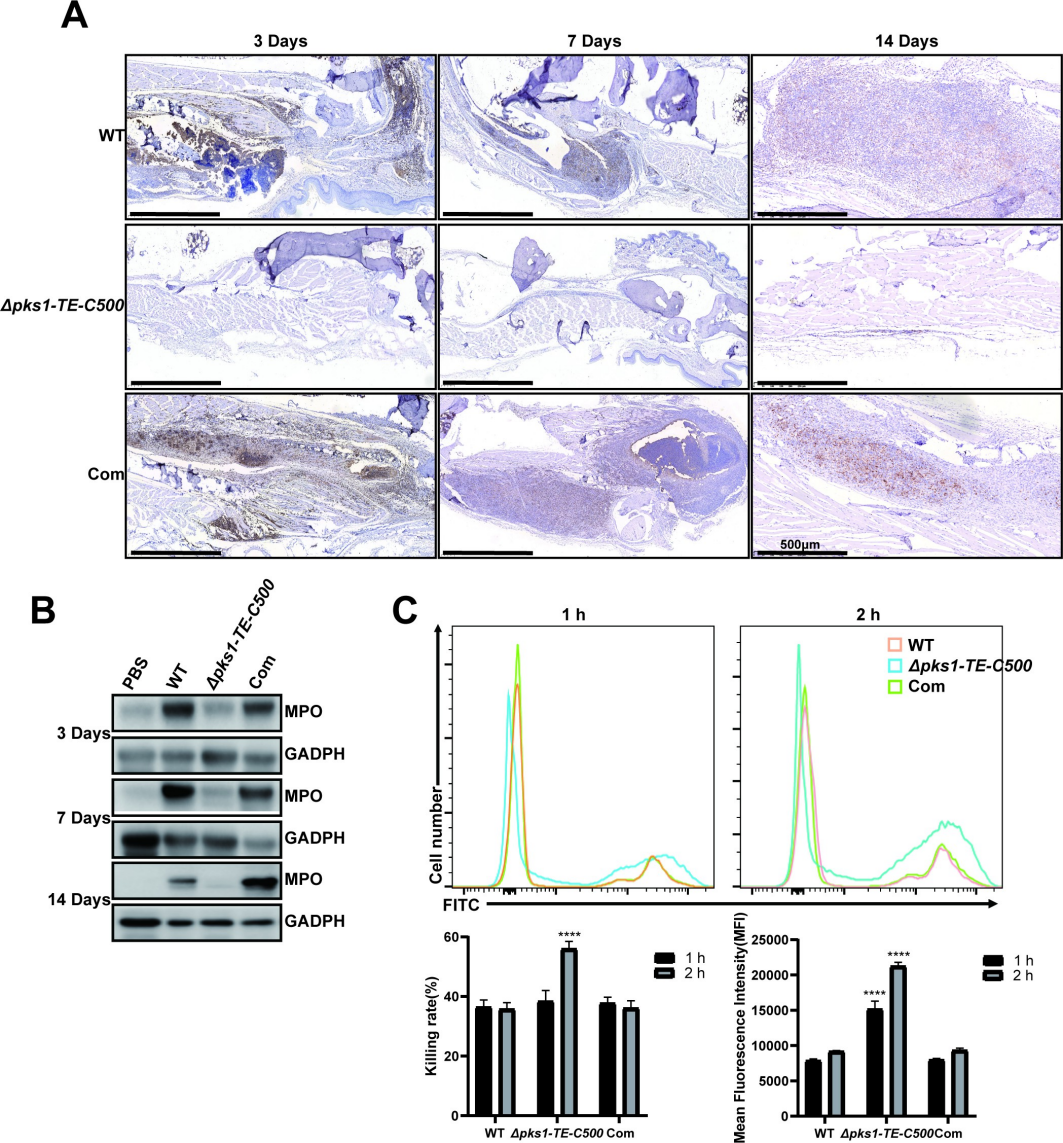
The 500 bp C-terminal of TE affects the recruitment of neutrophils in the footpads. The infiltration of neutrophils was detected in the footpads on the different days after injection. (A) Immunohistochemistry was performed to assess the expression of MPO in the footpads of infected mice. The *Δpks1-TE-C500* strain had a limited effect on neutrophil recruitment. (B) Western blotting was performed to detect MPO in footpad tissues. MPO was highly expressed in footpad tissues on the 3rd, 7th, and 14th day after injection with the wild-type and complemented strains. (C) After each strain was co-cultured with human blood-derived neutrophils for 1 h and 2 h, the phagocytosis rate was determined by flow cytometry, and the survival rate of conidia was measured by counting CFU on PDA. Statistical significance was determined by two-tailed t-test (****, P<0.0001). The *Δpks1-TE-C500* had higher phagocytosis and survival rate.

There were significantly fewer neutrophils in the footpads of mice injected with the mutant versus the wild-type or complemented strain when detected by immunohistochemistry or western blotting (**Fig 8A and 8B**). In neutrophil killing assay, each strain was co-cultured with human blood neutrophils for 1 h and 2 h, the phagocytosis rate was determined by flow cytometry. Through counting the CFU, our data confirmed that the mutant strain was more likely to be phagocytosed and killed by neutrophils, which were consistent with the findings of *in vitro* macrophage killing assay (**Fig 8C**).

## Discussion

PKSs in many organisms contain a C-terminal TE in their multidomain modules [24] and integrity of TE domain in *F*. *monophora* PKS seemed to be critical for melanin synthesis in our previous study [13]. TE domain in *F*. *monophora* PKS can be classified into TE16 family according to a previous report [25] that has oleoyl-ACP hydrolase (EC3.1.2.14) activity. The functions of the TE domain include termination of fatty acid synthesis, polyketide biosynthesis, and non-ribosomal peptide biosynthesis, known as thioesterase I. To verify the function of TE domain of PKS on *F*. *monophora* virulence, we constructed a C-terminal TE deletion mutant by knocking out the last 500 bp of AYO21_03016 (*pks1*) in this study. The consequences of metabolic abnormality in fungal growth, pathogenic traits and virulence are then examined *in vitro* and *in vivo* conditions. The TE-mediated pathogenicity has been demonstrated in some bacteria which have TEs belonging to different TE families. However, the pathogenesis of the TE domain on PKS has not been determined [26–28]. To the best of our knowledge, it is the first study to assess pathogenic effects of TE on fungal PKS.

PKS gene can regulate the development of conidia in *Beauveria bassiana* [29], *Pestalotiopsis microspore* [30] and *Chaitomium globosum* [31]. In *Pestalotiopsis fici*, PKS has been proved to be essential not only to protect spores from biological and abiotic stress, but also to the development of spore structure [32]. In this study, the *Δpks1-TE-C500* strain also shows defects in growth and the development of conidia demonstrated by smaller-sized, less, and delayed germination. Our results are similar to the reports found in other fungi after deletion of PKS [30,33–35]. Given the fact that *pks1* expression significantly decreased in the *Δpks1-TE-C500* strain, all these findings indicate that TE on *pks1* act as a trigger for growth and sporulation in *F*. *monophora*.

The reproduction and germination of conidia are the core components of survival in the environment, as well as the important determinants of pathogenicity [36]. For example, fungal conidia face various stresses in the human body, such as heat, salt, oxidative stress, osmotic pressure, nutritional deficiencies, and cytokine-mediated killing. In addition, melanin in fungi is also essential for the resistance to external stresses. Studies on *C*. *neoformans* and *F*. *pedrosoi* have shown that melanin can convert ferric iron to ferrous iron, thereby forming an iron-melanin complex to eliminate oxidative free radicals [37,38], which plausibly explains that the *Δpks1-TE-C500* strain is more sensitive to an increased sensitivity to H_2_O_2_ and NO *in vitro* during mycelial growth. We speculate that the loss of TE affects the synthesis of melanin, which may not effectively eliminate oxidative free radicals, resulting in the increase sensitivity to oxidative stress.

The stress adaptation not only helps fungi survive in extreme environments, but also plays an important role in anti-killing and replication in macrophages and neutrophils. In our study, viability and morphogenesis of the *Δpks1-TE-C500* within the macrophages are impaired and mutant cells were enclosed within the lysosomes of microphages. These results are consistent with the study in *Aspergillus fumigatus*, in which *pksP* (*alb1*) was required for defense of lysosomal fusion and intracellular killing [39]. Melanin is believed to decrease a pathogen’s resistance to the host antimicrobial mechanisms and influence the host immune response to infection [40]. Recent studies have demonstrated that calcium sequestration by DHN-melanin inhibits essential host effector pathways regulating phagosome biogenesis and prevents *A*. *fumigatus* conidia killing by phagocytes [41,42]. All these findings indicated that the loss of melanin is an important reason for the attenuated virulence of the *Δpks1-TE-C500* strain besides the defects in growth and germination mentioned above.

PKS regulates the biosynthesis of various secondary metabolites through the biosynthetic intermediates besides being a key enzyme for melanin formation [43]. Our findings indicate that the TE domain is required for *pks1* expression in *F*. *monophora* since down-regulated expression of each of remaining five enzymes in *pks1* after knocking out TE. Metabolomic profiles showed a significant increase on anti-oxidant glutathione (reduced form) or nicotinamide in *Δpks1-TE-C500* strain. The pathogenic impact of increased anti-oxidant glutathione and nicotinamide in *Δpks1-TE-C500* strain is unclear, but it could be an overall result from a downregulated *pks1*. Villarama *et al*. found that glutathione inhibits the formation of melanin through mediating the switch mechanism from eumelanin (L-DOPA pathway) to pheomelanin production [44], which could be used to explain the albino phenotype of *Δpks1-TE-C500* strain due to supplementation of L-tyrosine and L-DOPA did not restore melanin in *Δpks1-TE-C500* strain.

In addition to melanin biosynthesis, glutathione has anti-inflammatory effect by inhibiting the production of IL-6, IL-8, and TNF-α [45] and nicotinamide also shows potent inhibitory effects on proinflammatory cytokines from the host [46]. Ciebiada-Adamiec et al. found that nicotinamide inhibited antifungal activity against *Candida albicans*, *Trichophyton rubrum*, *and Trichophyton mentagrophytes* by decreasing pathogenesis-associated enzymatic activities [47]. These anti-inflammatory effects of fungal glutathione and nicotinamide might explain the mild footpad swelling and decreased inflammation in mice infected with the *Δpks1-TE-C500* mutant. Besides, there were some metabolites dropped sharply in mutant strain, including gossypol, cephalosporin_C, ydroxyspheroidenone, okenone and penitrem_D. Gossypol is an inhibitor of eukaryotic cells with an undetermined mode of action [48,49]. The relationship between its reduction *in vivo* and the pathogenicity of our mutant strain is still not clear, which deserves further investigation. An inverse relation was observed between the ability to synthesize cephalosporin C and the growth rate in *Cephalosporium acremonium* mutants [50]. However, the *Δpks1-TE-C500* strain in our study has a significantly reduced production of cephalosporin C and slow growth at the same time, suggesting a various relationship between cephalosporin C and growth in different strains. Studies on hydroxyspheroidenone, okenone and penitrem_D in fungi were limited and their function are still unclear.

The importance of neutrophils for the prevention and clearance of invasive fungal infection is widely recognized [51]. IL-17A and Th17 cells are considered to confer protection against fungi by recruitment via CXC chemokines [52]. In footpads injected with wild-type strain, neutrophils and Th17 were the main cell lineages found in the infected area during the early stage of experimental murine CBM, which is similar to increased Th17 cells found in the early-infected stage of *F*. *pedrosoi* [53]. In the mutant strain infection, we noted that Th17-related cytokines (IL-17A, IL-22) and neutrophil-related chemokines (CCL2, CCL3, CXCL1, CXCL2) are significantly reduced at the early stage of infection, which might be related to the absence of melanin and defects in growth and germination. However, the *pksP*/*alb1* mutant of *A*. *fumigatus* conidia promoted the production of pro-inflammatory cytokines [54], suggesting a variety of *pks1*-associated inflammatory effects among different fungi. In consistence of cytokines responses, in particular increased neutrophil-related chemokines, an abundant neutrophil infiltration is shown in the footpads of mice injected with the wild-type strain but not in mutant infected mice. These results indicate that the TE plays an important role in promoting the host’s inflammatory response and is essential for the virulence of *F*. *monophora*.

As a NTD, CBM is well-known for its resistance to most treatments and prone to recurrence [55]. Recent studies have shown that impaired fungal clearance in CBM could be related to inherent virulence traits of pathogens such as thermotolerance, cellularity with thick cell walls, cell adhesion, hydrophobicity and melanin [56–58]. Our data indicate that the knockout of TE affected the synthesis of melanin and virulence of *F*. *monophora*, which provides new insights into the pathogenic mechanism of CBM and new therapeutic development.

In summary, an albino *F*. *monophora* strain is successfully obtained by knocking out C-terminus of TE gene in this study. Several findings and conclusions can be drawn. First, disruption of TE catalytic active site (Ser-His-Asp triad) results in the melanin loss, decreases *pks1* gene expression and affects biosynthesis of secondary metabolites. Second, reduction of melanin or other secondary metabolites in mutant leads to slow growth, delays germination, and increases the sensitivities to environmental stresses such as oxidative stresses. Third, mutant strain has a compromised capacities to invade murine footpads and to induce inflammatory responses, and is easily killed by macrophages and neutrophils. These results suggest that C-terminal TE is essential in maintaining fully function of *pks1* and fungal pathogenicity of *F*. *monophora*.

## Supporting information

S1 Fig Conserved functional domains of AYO21_03016, AYO21_10638 andAYO21_03016 mutant proteins, analyzed by InterProScan 4.8 (starter ACP transacylase (SAT), β-ketoacyl synthase (KS), acetyl transferase (AT), dehydratase (DH), acyl carrier proteins (ACP) and thioesterase (TE)). The significant difference between AYO21_03016 and AYO21_10638 is the size of the TE domain, so in this study we knocked out the last 500 bp of AYO21_03016. After the last 500 bp of AYO21_03016 was deleted, the TE domain changed from the original 253 amino acids to 86 amino acids.
(TIF)

S2 Fig Sequence alignment of AYO21_03016 TE and other canonical TEs. Clustal Omega was used for the protein sequence alignment. The conserved catalytic residues of thioesterase (Ser—Asp—His) are labeled with red boxes. Note: PKS13-TE, accession number A0A098D6U0; AtCURS2-TE, accession number AGC95321; CcRADS2-TE, accession number ACM42403.
(TIF)

S3 Fig Effect of the gene knockout size on the efficiency of *F*. *monophora* transformation. The plasmid with knockout of the terminal 500 bp of the *pks1* gene (*pks1-TE*) or whole gene (*pks1*) was transformed to *A*. *tumefaciens* strain EHA105. The pre-induced EHA105 cells and spores were mixed and co-cultured at the same time. Transformants were selected on PDA plates with hygromycin B (50 μg/ml) and cefotaxime (200 μM) and incubated at room temperature for 7 days. The number of transformants and the ratio of positive transformants (determined by phenotype) under each condition were calculated. Statistical significance was determined by two-tailed t-test (**P<0.01; ***P<0.001).
(TIF)

S4 Fig PDA plates with tricyclazole (50 μg/ml, second row) inoculated for 14 days at 25°C with the wild-type (left side in the plate) and *pks1* TE mutant (right side in the plate) strains together. Plates with L-DOPA (50 μg/ml, third row) or L-tyrosine (50 μg/ml, fourth row) inoculated with the wild-type and mutant strains did not show color change. The mutant with L-Dopa or with L-tyrosine, similar to the control group (first row).
(TIF)

S5 Fig Loss of *pks1* TE affects conidial germination. Each strain was cultured in SDB for 0, 12, 24, and 48 h, and the germination of the *Δpks1-TE-C500* was slower.
(TIF)

S6 Fig Volcano map of RNA-sequencing analysis. Differential gene volcano map revealed that the *Δpks1-TE-C500* group and the PBS group had a low number of differentially expressed genes.
(TIF)

S7 Fig KEGG pathway enrichment analysis and GO analysis. The results indicated that the wild-type strain could induce inflammation, immune response and neutrophil chemotaxis (black arrow).
(TIF)

S1 Table Primers used in this study. (DOCX)

S2 Table *pks1*-TE-500 KO vs WT or Com. (metabolomics). (XLSX)

S1 Data The numerical data used in all figures. (XLSX)
