# Supplementary figures and images for: Deletion C-terminal thioesterase abolishes melanin biosynthesis, affects metabolism and reduces the pathogenesis of Fonsecaea monophora
Source: PLoS Negl Trop Dis. 2022 Jun 13;16(6):e0010485. doi: 10.1371/journal.pntd.0010485 (PMC9255740; doi:10.1371/journal.pntd.0010485)

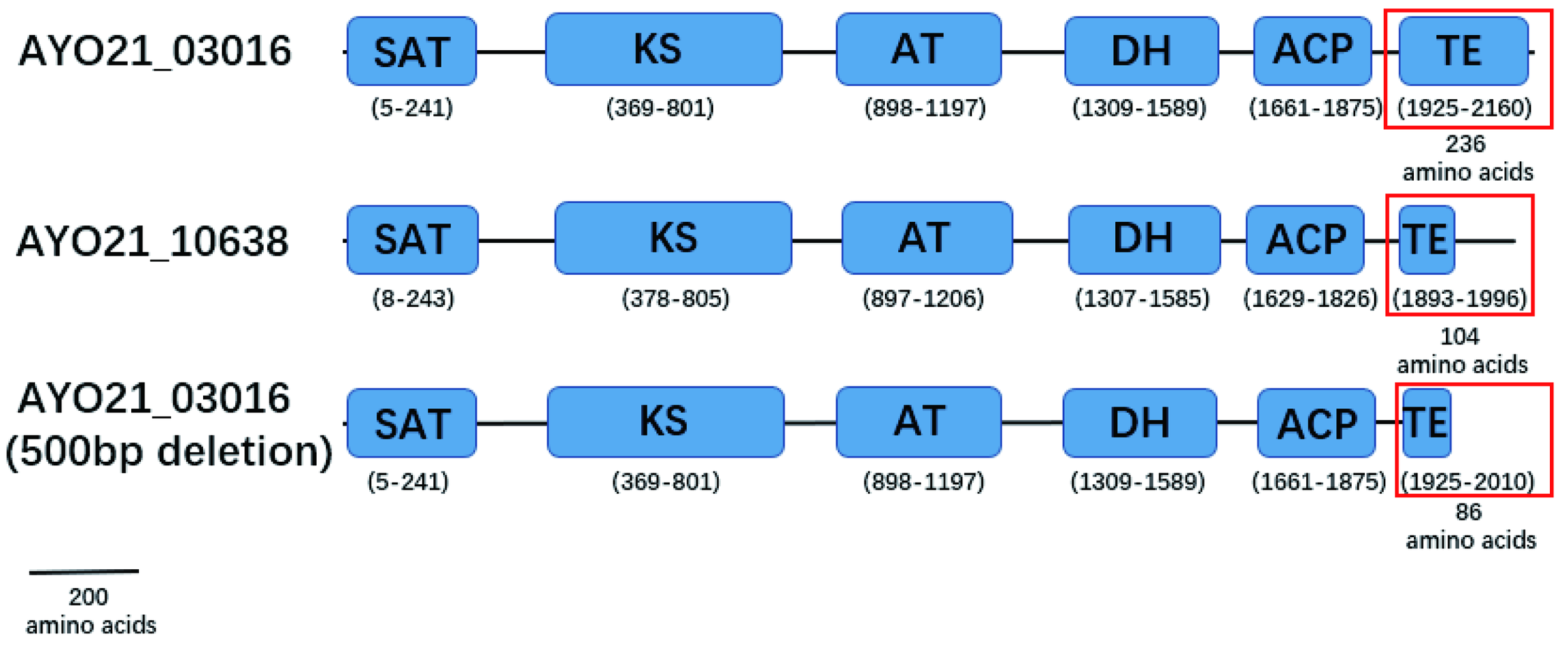

Supplement: S1 Fig — The significant difference between AYO21_03016 and AYO21_10638 is the size of the TE domain, so in this study we knocked out the last 500 bp of AYO21_03016. After the last 500 bp of AYO21_03016 was deleted, the TE domain changed from the original 253 amino acids to 86 amino acids. (TIF) [file pntd.0010485.s001.tif]

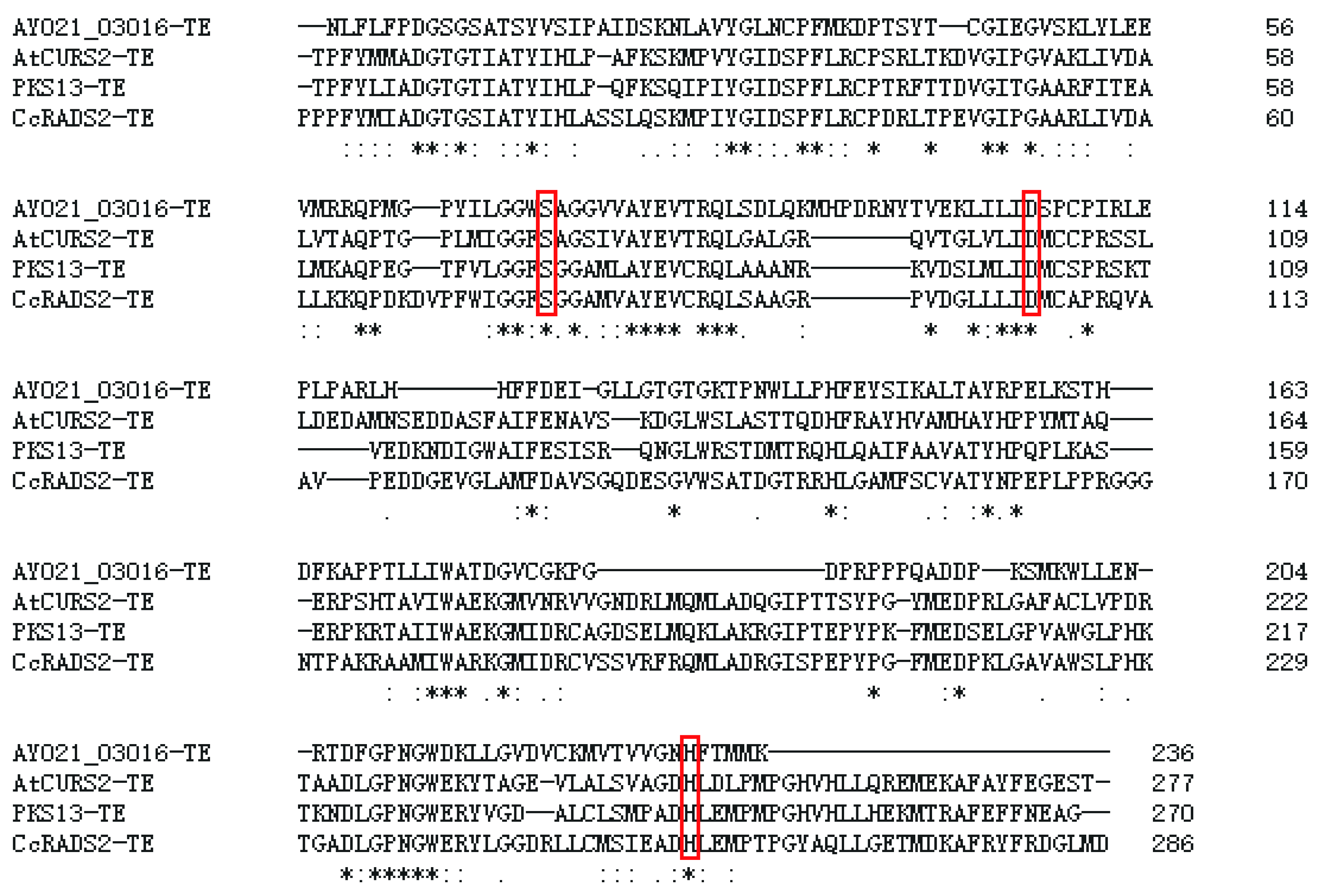

Supplement: S2 Fig — Clustal Omega was used for the protein sequence alignment. The conserved catalytic residues of thioesterase (Ser—Asp—His) are labeled with red boxes. Note: PKS13-TE, accession number A0A098D6U0; AtCURS2-TE, accession number AGC95321; CcRADS2-TE, accession number ACM42403. (TIF) [file pntd.0010485.s002.tif]

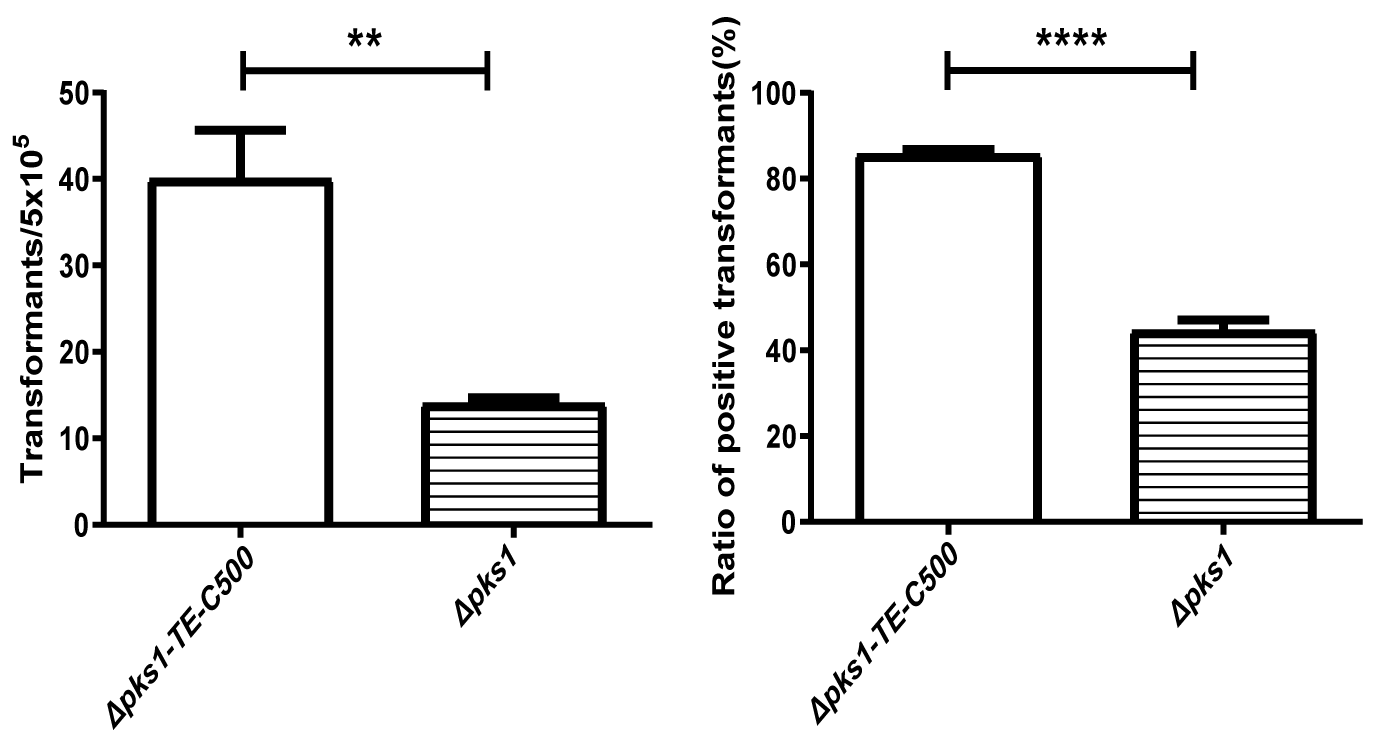

Supplement: S3 Fig — The plasmid with knockout of the terminal 500 bp of the pks1 gene (pks1-TE) or whole gene (pks1) was transformed to A. tumefaciens strain EHA105. The pre-induced EHA105 cells and spores were mixed and co-cultured at the same time. Transformants were selected on PDA plates with hygromycin B (50 μg/ml) and cefotaxime (200 μM) and incubated at room temperature for 7 days. The number of transformants and the ratio of positive transformants (determined by phenotype) under each condition were calculated. Statistical significance was determined by two-tailed t-test (**P<0.01; ***P<0.001). (TIF) [file pntd.0010485.s003.tif]

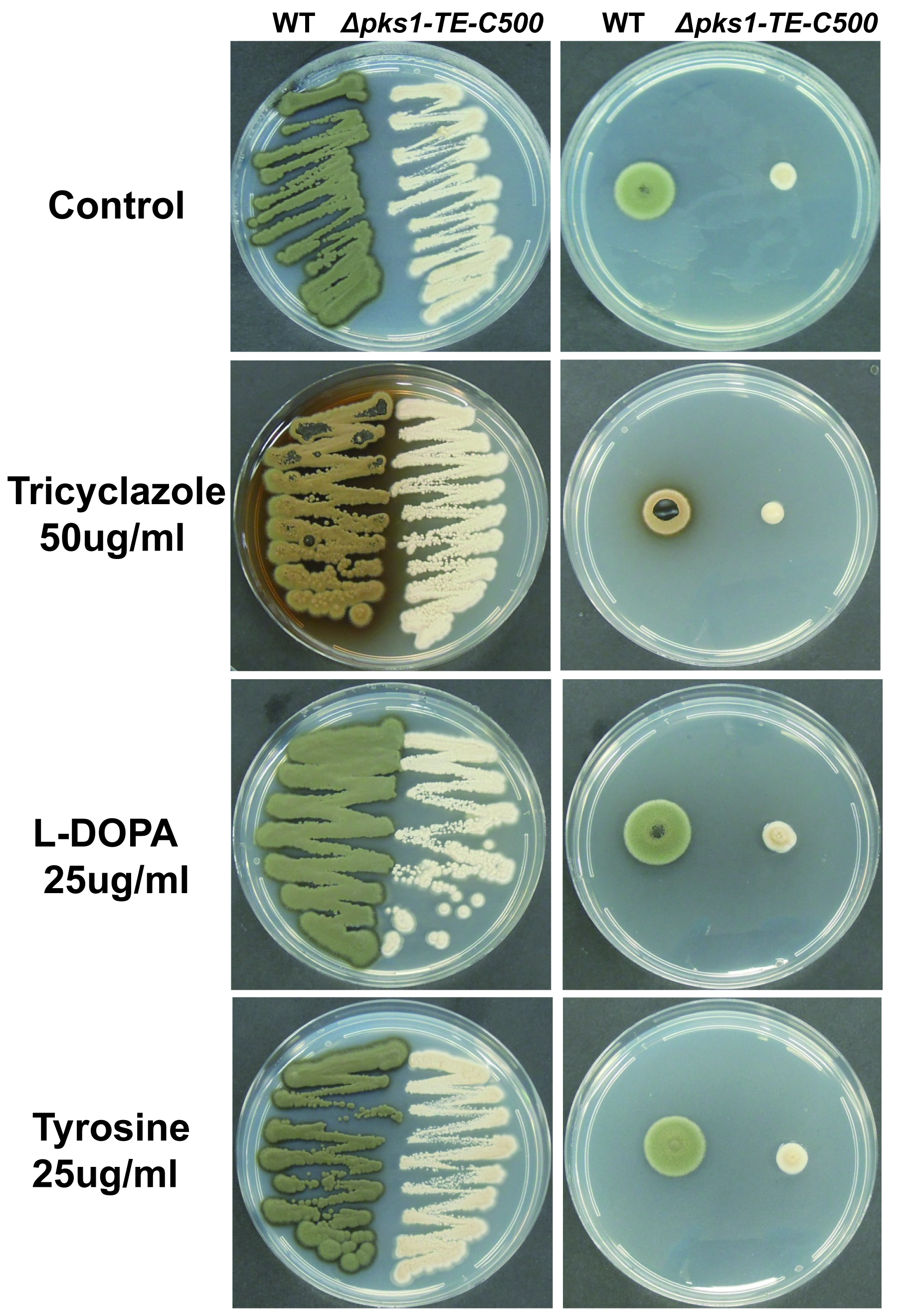

Supplement: S4 Fig — Plates with L-DOPA (50 μg/ml, third row) or L-tyrosine (50 μg/ml, fourth row) inoculated with the wild-type and mutant strains did not show color change. The mutant with L-Dopa or with L-tyrosine, similar to the control group (first row). (TIF) [file pntd.0010485.s004.tif]

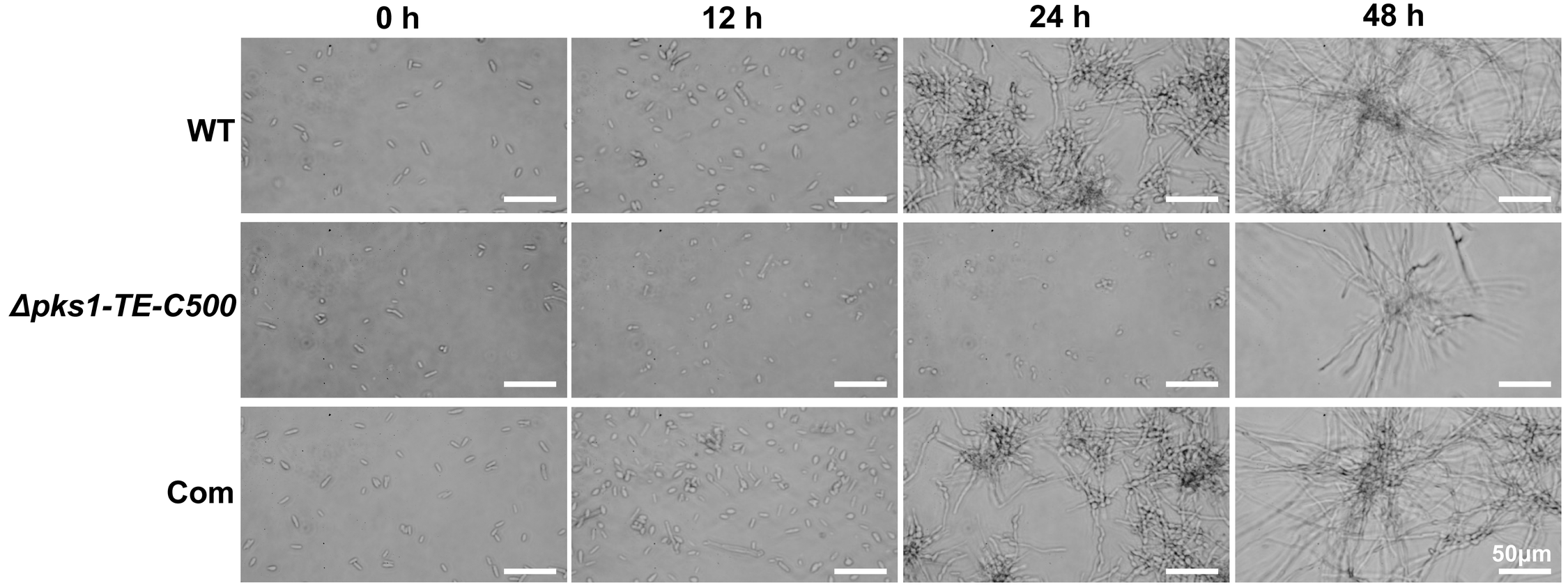

Supplement: S5 Fig — Each strain was cultured in SDB for 0, 12, 24, and 48 h, and the germination of the Δpks1-TE-C500 was slower. (TIF) [file pntd.0010485.s005.tif]

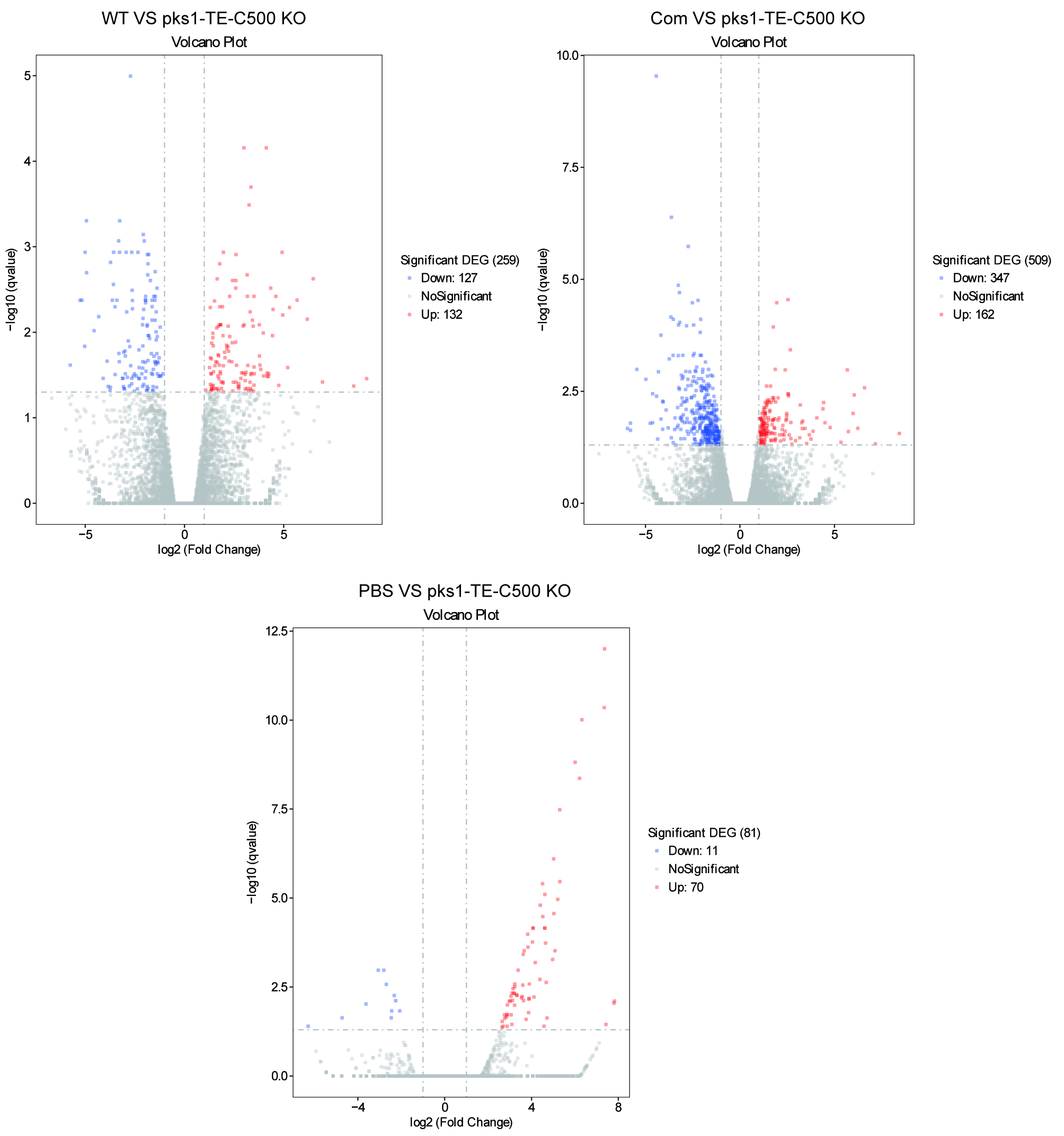

Supplement: S6 Fig — Differential gene volcano map revealed that the Δpks1-TE-C500 group and the PBS group had a low number of differentially expressed genes. (TIF) [file pntd.0010485.s006.tif]

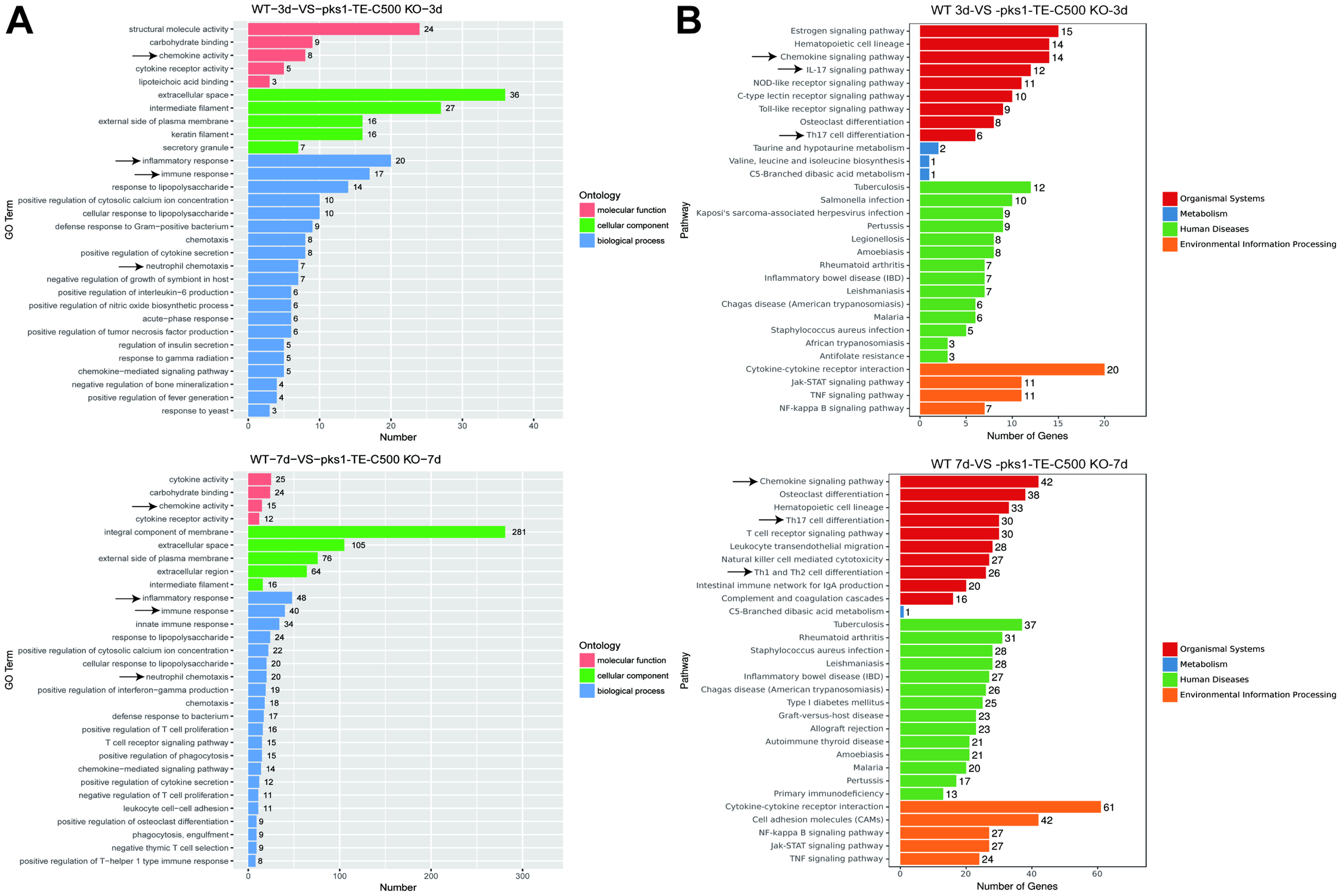

Supplement: S7 Fig — The results indicated that the wild-type strain could induce inflammation, immune response and neutrophil chemotaxis (black arrow). (TIF) [file pntd.0010485.s007.tif]
