# Supplementary material for: Deletion C-terminal thioesterase abolishes melanin biosynthesis, affects metabolism and reduces the pathogenesis of Fonsecaea monophora
Source: PLoS Negl Trop Dis. 2022 Jun 13;16(6):e0010485. doi: 10.1371/journal.pntd.0010485 (PMC9255740; doi:10.1371/journal.pntd.0010485)
Supplement: S1 Table — (DOCX) [file pntd.0010485.s008.docx]

**TABLE S1** Primers used in this study

| Primer Name | Sequence (5'-3') |
| --- | --- |
| primers 1 | aaaacgtccgcaatgCGCGCGCGtgttattaagttgtcta |
| primers 2 | Agccgattttgaaaccgcggtgatcacaggcagca |
| primers 3 | cattgcggacgtttttaatgCTCGAGtactgaattaacgccgaat |
| primers 4 | cggaggccatggatgcgatcgctgcggccgatctt |
| *pks1*-TE arm1. FOR | gtttttaatgCTCGAACAAACTACTCGTCAAAGTATGCATATGC |
| *pks1*-TE arm1. REV | taattcagtaCTCGAGGTGACCTTTGACATGCTCATTGTCAC |
| *pks1*-TE arm2. FOR | atgattacgaattcgCGATCAAGATCAACTTCTCGACCGTGTAGT |
| *pks1*-TE arm2. REV | cgactctagaggatcATGCCCAAGCCCAAGG |
| *pks1-*Com arm3. FOR | atgattacgaattcgCTAAGACAAAAGAGCCTCGCGGATG |
| *pks1-*Com arm3. REV | cgactctagaggatcATGCCCAAGCCCAAGG |
| *pks1*-TE. FOR | CGTGTCCGTCAACCCCAGCC |
| *pks1*-TE. REV | CTTGCCATCTGTCTGGCTTT |
| q-SAT-F | CTGCACTGGATGCGTTTCAC |
| q-SAT-R | TATGGCGCGTAGATGGACAC |
| q-KS-F | GTACCAACCACTCGGCAGAA |
| q-KS-R | TTGCAGGGCACATTGCATTC |
| q-AT-F | TGCCTCTGATTGACGGATCG |
| q-AT-R | AGACGGGTGAGAGCCATTTG |
| q-DH-F | GGCTGACTTCACCGTTACCA |
| q-DH-R | TTCCACTCGTCCTTCCAAGC |
| q-ACP-F | CTGGCCGAGATTACCGAGAC |
| q-ACP-R | AAATGTCCGAGCGATGGGTT |
| q-TE-F | ACCCCAAGAGCATGAAGTGG |
| q-TE-R | ACACATCAACGCCCAAAAGC |
